# Supplementary material for: A simple low-latency real-time certifiable quantum random number generator
Source: Nat Commun. 2021 Feb 24;12:1056. doi: 10.1038/s41467-021-21069-8 (PMC7904850; doi:10.1038/s41467-021-21069-8)
Supplement: Supplementary file 1 — Supplementary Information [file 41467_2021_21069_MOESM1_ESM.pdf]

Supplementary Information: A Simple Low-Latency Real-Time Certifiable Quantum Random Number Generator

Yanbao Zhang,[1,2] Hsin-Pin Lo,[1] Alan Mink,[3] Takuya Ikuta,[1]
Toshimori Honjo,[1] Hiroki Takesue,[1] and William J. Munro[1,2]

[1]*NTT Basic Research Laboratories, NTT Corporation, 3-1 Morinosato-Wakamiya, Atsugi, Kanagawa 243-0198, Japan*
[2]*NTT Research Center for Theoretical Quantum Physics, NTT Corporation, 3-1 Morinosato-Wakamiya, Atsugi, Kanagawa 243-0198, Japan*
[3]*National Institute of Standards and Technology, Gaithersburg, MD 20899, USA*

We investigate a simple scheme for randomness generation. In the ideal case, we prepare and measure the quantum state of a single photon (a qubit) where information can be encoded either in the polarization or time-bin degree of freedom. At each trial the measurement is along one of two bases, the X- and Z-bases, which are randomly selected with probabilities P_X and $P_Z(= 1 - P_X)$. Under each basis, there are two possible measurement outcomes $+1$ or -1. Since the prepared qubit state is allowed to be arbitrary, the measurements along the X- and Z-bases can be described without loss of generality by the operators $\sigma_X = \boldsymbol{\sigma} \cdot \mathbf{n}_X$ and $\sigma_Z = \boldsymbol{\sigma} \cdot \mathbf{n}_Z$, where $\mathbf{n}_X$ and $\mathbf{n}_Z$ are two unit vectors of three dimensions and $\boldsymbol{\sigma} = (\sigma_1, \sigma_2, \sigma_3)$ is the Pauli-matrix vector. In this work we assume that the measurements on the qubit are projective, but as remarked later when constructing the classical and quantum models, our method can deal with general measurements that can be decomposed into convex combinations of the assumed projective measurements. Ideally the two measurements along the X- and Z-bases are mutually unbiased. For example, one is the σ_1-measurement and the other is the σ_3-measurement. In this case the angle θ between the two directions $\mathbf{n}_X$ and $\mathbf{n}_Z$ is $\pi/2$. In practice, we need to consider imperfections in the implementation of the above scheme. The imperfections to be considered depend on the specific implementation. In our experiment, there are the following three types of imperfections. First, the two measurement bases can be misaligned such that $\theta = \pi/2 + \Delta\theta$, where the value of $\Delta\theta$ is not exactly known but can be bounded as $|\Delta\theta| \leq \delta$. Second, single-photon sources are not easily accessible in practice; instead weak optical pulses which have multiphoton components are employed. We note that for the setups considered in this work, the measurement operators are block-diagonal with respect to various photon-number subspaces. Hence, without loss of generality the weak optical pulse used can be assumed to be dephased such that there is no coherence between different photon-number components. Third, the probabilities P_X and P_Z of selecting the two bases are not exactly known. Furthermore, in the adversarial scenario the adversary could manipulate these imperfections.

The supplementary information is structured as follows. In Supplementary Note 1 and Supplementary Note 2 we provide details of how to consider the above adversarial imperfections for certifiable randomness generation with respect to the classical and quantum side information, respectively, of an adversary known as Eve. For this purpose, we need only a lower bound $q_{1,\mathrm{lb}}$ on the probability of emitting a single photon from a practical photon source, an upper bound δ on the misalignment angle $|\Delta\theta|$ between the two bases in the single-photon subspace, and a lower bound τ_{lb} as well as an upper bound τ_{ub} on the imbalance $\tau = (P_X - P_Z)/2$. For simplicity, we just call δ the misalignment angle in everywhere else of the supplementary information. We note that a lower bound on the probability of emitting a single photon relative to emitting any positive number of photons rather than a lower bound on the absolute probability of emitting a single photon is sufficient (see the treatment in Supplementary Note 3 B about the imperfections in the photon source). In Supplementary Note 3, we show that the adversarial imperfections in our experiment can be characterized by the above parameters $q_{1,\mathrm{lb}}$, δ, τ_{lb} and τ_{ub}. In Supplementary Note 4, we present the details of our security analysis (see Box 1 for an overview) and the corresponding results. These results demonstrate that with the security-analysis method developed in Supplementary Note 1 and Supplementary Note 2 we can realize a continuously-operating, high-security, high-speed and real-time quantum random number generator (QRNG).

Supplementary Note 1. Probability estimation for our experiment

In this supplementary note, we present the details of how to implement the framework of probability estimation for certifying randomness against classical side information [1, 2]. We note that for device-independent randomness generation, the excellent finite-data efficiency of probability estimation has been demonstrated in Refs. [1, 2]. Here we would like to apply probability estimation for low-latency randomness generation with partially characterized quantum devices. Below we construct the classical model for each trial and the corresponding probability estimation factors (PEFs) for the experiment of our interest. We emphasize that probability estimation is a general framework

for certifying randomness against classical side information; however, the implementation of this framework is case-dependent.

A. Construction of the classical model

To construct the classical model for a trial, we first temporarily assume that Eve's classical side information about the state is independent of Eve's partial knowledge of the input and measurement at the trial. Then we remark and explain that the classical model in the general case where Eve's classical side information about the state is classically correlated with Eve's partial knowledge of the input and measurement can be easily obtained as the convex closure of the model $\mathcal{C}$ constructed with the above independence assumption. The model $\mathcal{C}$ can be constructed by considering all the possible combinations of Eve's classical side information about the state and Eve's partial knowledge of the input and measurement, which is detailed in the following three steps:

Step 1. *We consider the case where at each trial the photon source emits a single photon with probability* 1 *and the probabilities P_X and P_Z of selecting the X- and Z-bases are fixed.* Since each measurement has two possible outcomes $+1$ or -1 and the probabilities P_X and P_Z are fixed, the set of probability distributions of the input (basis choice, denoted by I) and output (measurement outcome, denoted by O) achievable at each trial is fully determined by the region accessible by the expectation values $\langle\sigma_X\rangle$ and $\langle\sigma_Z\rangle$ of the measurements $\sigma_X = \boldsymbol{\sigma}\cdot\mathbf{n}_X$ and $\sigma_Z = \boldsymbol{\sigma}\cdot\mathbf{n}_Z$ on an arbitrary qubit state. So, we need only to characterize the region accessible by $\langle\sigma_X\rangle$ and $\langle\sigma_Z\rangle$, which is detailed in the next paragraph.

Given two arbitrary unit vectors $\mathbf{n}_X$ and $\mathbf{n}_Z$, we have that $(\mathbf{n}_X+\mathbf{n}_Z)\cdot(\mathbf{n}_X-\mathbf{n}_Z)=0$. Hence, the two Pauli matrices $\sigma_+ = \boldsymbol{\sigma}\cdot\mathbf{n}_+$ and $\sigma_- = \boldsymbol{\sigma}\cdot\mathbf{n}_-$, where $\mathbf{n}_+ = (\mathbf{n}_X+\mathbf{n}_Z)/\sqrt{2+2\mathbf{n}_X\cdot\mathbf{n}_Z}$ and $\mathbf{n}_- = (\mathbf{n}_X-\mathbf{n}_Z)/\sqrt{2-2\mathbf{n}_X\cdot\mathbf{n}_Z}$, are orthogonal, that is, $\mathrm{Tr}(\sigma_+^\dagger\sigma_-)=0$. So, there exists another Pauli matrix σ' such that the set $\{\mathbb{1}_2, \sigma_+, \sigma_-, \sigma'\}$, where $\mathbb{1}_2$ is the identity matrix of 2×2, is an orthonormal basis for the Hilbert space of a qubit. Then, in view of the fact that the purity $\mathrm{Tr}(\rho^2)$ of an arbitrary qubit state ρ cannot be larger than 1, we obtain the inequality

$$\langle\sigma_+\rangle^2 + \langle\sigma_-\rangle^2 \leq 1. \tag{1}$$

Therefore, if the angle between the two directions $\mathbf{n}_X$ and $\mathbf{n}_Z$ is fixed to be θ, the region $\mathcal{R}_{1,\theta}$ accessible by $\langle\sigma_X\rangle$ and $\langle\sigma_Z\rangle$ is described as

$$\frac{(\langle\sigma_X\rangle+\langle\sigma_Z\rangle)^2}{2+2\cos(\theta)} + \frac{(\langle\sigma_X\rangle-\langle\sigma_Z\rangle)^2}{2-2\cos(\theta)} \leq 1. \tag{2}$$

We observe that the region $\mathcal{R}_{1,\theta}$ has a shape of a filled ellipse, so it is convex. In practice the angle θ cannot be precisely characterized and can vary from trial to trial; however, by calibration we can ensure a bound on the deviation such that $|\theta-\pi/2|\leq\delta$ at each trial. Then, the region $\mathcal{R}_1$ accessible by $\langle\sigma_X\rangle$ and $\langle\sigma_Z\rangle$ becomes the union of the convex regions $\mathcal{R}_{1,\theta}$ accessible by all θ satisfying $|\theta-\pi/2|\leq\delta$. Since the angle θ takes a value from a convex set and for each possible θ the region $\mathcal{R}_{1,\theta}$ is convex, so is the region $\mathcal{R}_1$. Specifically, in the first quadrant where $\langle\sigma_X\rangle\geq 0$ and $\langle\sigma_Z\rangle\geq 0$, the boundary of the region $\mathcal{R}_1$ is described as follows:

$$\begin{cases} \text{when } 0\leq\langle\sigma_X\rangle\leq\sin(\delta), \langle\sigma_Z\rangle = 1; \\ \text{when } \sin(\delta)<\langle\sigma_X\rangle<1, \frac{(\langle\sigma_X\rangle+\langle\sigma_Z\rangle)^2}{2+2\sin(\delta)} + \frac{(\langle\sigma_X\rangle-\langle\sigma_Z\rangle)^2}{2-2\sin(\delta)} = 1; \\ \text{when } \langle\sigma_X\rangle = 1, 0\leq\langle\sigma_Z\rangle\leq\sin(\delta). \end{cases} \tag{3}$$

By symmetries, we can also obtain the boundary of the region $\mathcal{R}_1$ in the other quadrants, which is illustrated in Supplementary Figure 1.

We now can remark on the possibility of dealing with positive operator-valued measures (POVMs). Suppose that the two measurement directions $\mathbf{n}_X$ and $\mathbf{n}_Z$ are in the X-Z plane of the Bloch sphere. Without loss of generality, let $\mathbf{n}_X = (\cos(\theta_X), \sin(\theta_X))$ and $\mathbf{n}_Z = (\sin(\theta_Z), \cos(\theta_Z))$, where $|\theta_X|\leq\delta_X$, $|\theta_Z|\leq\delta_Z$ and $\delta_X+\delta_Z\leq\delta$ in order to satisfy the bound on the misalignment. Then, the expectation values $(\langle\boldsymbol{\sigma}\cdot\mathbf{n}_X\rangle, \langle\boldsymbol{\sigma}\cdot\mathbf{n}_Z\rangle)$ are in the region $\mathcal{R}_1$. Because the region $\mathcal{R}_1$ is convex, the expectation values $(\langle M_X\rangle, \langle M_Z\rangle)$ of $M_X = \sum_k \omega_{X,k}\boldsymbol{\sigma}\cdot\mathbf{n}_{X,k}$ and $M_Z = \sum_l \omega_{Z,l}\boldsymbol{\sigma}\cdot\mathbf{n}_{Z,l}$, where for all k (l), $\omega_{X,k}\geq 0$ and $|\theta_{X,k}|\leq\delta_X$ ($\omega_{Z,l}\geq 0$ and $|\theta_{Z,l}|\leq\delta_Z$), and $\sum_k\omega_{X,k} = \sum_l\omega_{Z,l} = 1$, are also in the region $\mathcal{R}_1$. In other words, if we randomly choose to perform the POVM $\{(\mathbb{1}_2+M_X)/2, (\mathbb{1}_2-M_X)/2\}$ or $\{(\mathbb{1}_2+M_Z)/2, (\mathbb{1}_2-M_Z)/2\}$ on a qubit, the resulting distribution of the output O conditional on the input I is in the set determined by the convex region $\mathcal{R}_1$. Therefore, in our construction of the classical model not only the assumed projective measurements but also the POVMs that can be decomposed into convex combinations of these projective

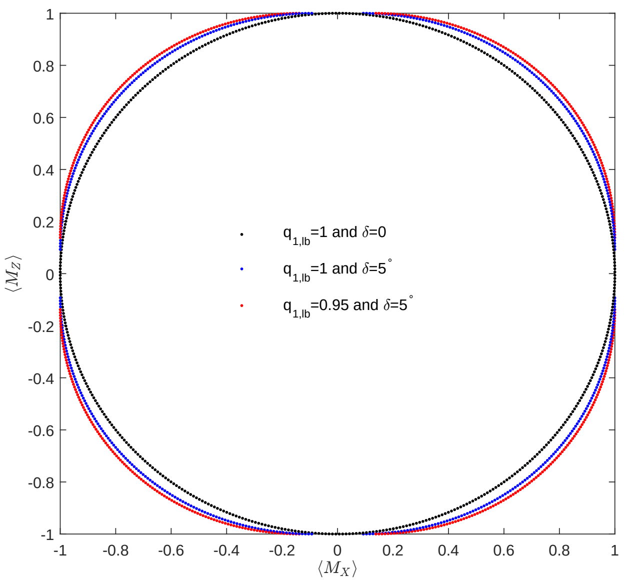

Supplementary Figure 1: Regions accessible by the expectation values $\langle M_X\rangle$ and $\langle M_Z\rangle$ under different values for the single-photon probability bound $q_{1,\mathrm{lb}}$ and the misalignment angle δ. Specifically, given the values of $q_{1,\mathrm{lb}}$ and δ the accessible region is inside of the corresponding boundary (the dotted curve). Note that when $q_{1,\mathrm{lb}} = 1$ (the single-photon case), the measurement operators can be represented by two Pauli matrices σ_X and σ_Z.

measurements are allowed. We emphasize that the set of POVMs considered above does not include all the possible POVMs that are close to the ideal projective measurement σ_1 or σ_3. Also, our current method does not allow Eve to hold purifications of POVMs, as it assumes that Eve can access classical side information but *not* quantum side information about the measurement performed at a trial. How to completely handle general POVMs with our method deserves further investigation in future work.

Step 2. *We consider the case where at each trial the probability of emitting a single photon from the photon source is at least $q_{1,lb}$ and the probabilities of selecting the X- and Z-bases are fixed.* For the same reason as in Step 1, we need only to characterize the region accessible by the expectation values at the two measurement bases. Recall that in the single-photon subspace the two measurements are described by Pauli operators σ_X and σ_Z, while in the general case the two measurements, denoted by M_X and M_Z, may be difficult to be exactly specified but are always block-diagonal with respect to various photon-number subspaces. Because of the block-diagonal structure of M_X and M_Z, the expectation values $\langle M_X\rangle$ and $\langle M_Z\rangle$ can be expressed as convex combinations of those expectation values conditional on a varying number of photons emitted from the source. In Step 1, we already characterize the region $\mathcal{R}_1$ accessible by the expectation values $\langle M_X\rangle$ and $\langle M_Z\rangle$ conditionally on the emission of a single photon at a trial. Moreover, since each measurement has two possible outcomes $+1$ or -1, the region $\mathcal{R}_{\neq 1}$ accessible by $\langle M_X\rangle$ and $\langle M_Z\rangle$ conditionally on the emission of multiple photons or zero photon at a trial is at most as large as the square $\mathcal{R}_s \doteq \{-1 \le \langle M_X\rangle \le 1, -1 \le \langle M_Z\rangle \le 1\}$. For the model construction, we set $\mathcal{R}_{\neq 1} = \mathcal{R}_s$. That is, when the source emits multiple photons or zero photon we constrain the accessible region $\mathcal{R}_{\neq 1}$ in a device-independent way, regardless of the state prepared or measurements performed. Given that the single-photon probability is at least $q_{1,\mathrm{lb}}$, the region $\mathcal{R}$ accessible by $\langle M_X\rangle$ and $\langle M_Z\rangle$ is thus described as

$$\begin{aligned}\mathcal{R} &= \bigcup_{1\ge\omega\ge q_{1,\mathrm{lb}}} \left(\omega\mathcal{R}_1 + (1-\omega)\mathcal{R}_s\right)\\ &= q_{1,\mathrm{lb}}\mathcal{R}_1 + (1-q_{1,\mathrm{lb}})\mathcal{R}_s.\end{aligned} \tag{4}$$

Here the equality in the second line of the above equation is due to the fact that $\mathcal{R}_1 \subseteq \mathcal{R}_s$. Since both regions $\mathcal{R}_1$ and $\mathcal{R}_s$ are convex and $\mathcal{R}_1 \subseteq \mathcal{R}_s$, the region $\mathcal{R}$, as illustrated in Supplementary Figure 1, is convex. We remark that Eq. (4) implies the statement in the main text that the classical model $\mathcal{C}$ can be expressed as a convex combination of sub-models constructed conditionally on a varying number of photons emitted from the source.

Before moving to the next step, we recall that the expectation values $\langle M_X\rangle$ and $\langle M_Z\rangle$ are implicitly conditional on Eve's classical side information. Therefore, by setting $\mathcal{R}_{\neq 1} = \mathcal{R}_s$ we pessimistically allow perfect correlations between

Eve's classical side information and all the possible measurement outcomes obtained when the source emits multiple photons or zero photon. In this way, we choose to not certify the randomness contributed by these outcomes. As a consequence, our security analysis is robust against photon-number splitting attacks.

Step 3. *We consider the general case where at each trial the probability of emitting a single photon from the photon source is at least $q_{1,lb}$ and the probabilities P_X and P_Z of selecting the X- and Z-bases are not exactly known but bounded such that $P_X + P_Z = 1$ and the imbalance $\tau = (P_X - P_Z)/2$ satisfies $\tau_{lb} \leq \tau \leq \tau_{ub}$.* The set of input distributions is thus given as

$$\mathcal{S} = \{\mathbb{P}(I) : \tau_{\mathrm{lb}} \leq (\mathbb{P}(I = X) - \mathbb{P}(I = Z))/2 \leq \tau_{\mathrm{ub}} \text{ and } \mathbb{P}(I = X) + \mathbb{P}(I = Z) = 1\}. \tag{5}$$

It is easy to see that $\mathcal{S}$ is a closed interval and so is convex. From Step 2, we know that the set of probability distributions of the output O conditional on the input I is characterized as follows:

$$\mathcal{C}_{O|I} = \Big\{\mathbb{P}(O|I) : \text{for each } i = X \text{ or } Z \text{ and } o = +1 \text{ or } -1, \mathbb{P}(O = o|I = i) = \frac{1 + o\langle M_i\rangle}{2}, \text{ where } \big(\langle M_X\rangle, \langle M_Z\rangle\big) \in \mathcal{R}\Big\}. \tag{6}$$

The set $\mathcal{C}_{O|I}$ is convex as each member of $\mathcal{C}_{O|I}$ is obtained from a member of the convex set $\mathcal{R}$ by a linear transformation. To obtain the joint distribution of I and O, we consider an arbitrary combination of the input distribution $\mathbb{P}(I) \in \mathcal{S}$ and the input-conditional distribution of outputs $\mathbb{P}(O|I) \in \mathcal{C}_{O|I}$. Accordingly, the classical model $\mathcal{C}$ (that is, the set of distributions of I and O achievable at a trial) is given as

$$\mathcal{C} = \Big\{\mathbb{P}(I, O) : \mathbb{P}(I) \in \mathcal{S}, \mathbb{P}(O|I) \in \mathcal{C}_{O|I}, \text{ and for each } i \text{ and } o, \mathbb{P}(I = i, O = o) = \mathbb{P}(I = i)\mathbb{P}(O = o|I = i)\Big\}. \tag{7}$$

The model $\mathcal{C}$ is convex because both sets $\mathcal{S}$ and $\mathcal{C}_{O|I}$ are convex.

We emphasize that to construct the classical model $\mathcal{C}$, we exploit only the fact that the measurements M_X and M_Z are block-diagonal with respect to various photon-number subspaces, and we do not use any information about the normalized state in each photon-number subspace. The measurement operators and the distribution of photon numbers emitted from the source, as well as the input distribution, may depend on some auxiliary degrees of freedom, such as spatial mode, which are not used for encoding information. If Eve can access these auxiliary degrees of freedom such as by a side-channel attack, Eve could be able to manipulate the measurements performed and the state prepared. In this case, we take advantage of the fact that in practice the measurements are block-diagonal with respect to various states for the auxiliary degrees of freedom manipulable by Eve (see the Methods section of the main text for detailed explanations). Therefore, as long as the adversarial imperfections are characterized by the parameters $(q_{1,\mathrm{lb}}, \delta, \tau_{\mathrm{lb}}, \tau_{\mathrm{ub}})$, the classical model $\mathcal{C}$ constructed as above, as well as the PEFs constructed below, is valid, meaning that our security analysis is robust against such side-channel attacks.

We finally remark that in the general case where Eve's classical side information about the state is classically correlated with Eve's partial knowledge of the input and measurement at a trial, the joint distribution of the input I and output O of the trial (conditional on the classical side information E implicitly) can be written as

$$\mathbb{P}(I, O) = \sum_{\lambda} \omega_{\Lambda=\lambda} \mathbb{P}^{\Lambda=\lambda}(I, O), \tag{8}$$

where for each λ, $\omega_{\Lambda=\lambda} \geq 0$, and $\sum_{\lambda} \omega_{\Lambda=\lambda} = 1$. Here, the random variable Λ characterizes the classical correlation between Eve's classical side information about the state and Eve's partial knowledge of the input and measurement, and for each possible value $\Lambda = \lambda$, the joint distribution $\mathbb{P}^{\Lambda=\lambda}(I, O)$ is in the model $\mathcal{C}$ of Eq. (7). Therefore, the classical model for the trial will become the convex closure of the classical model $\mathcal{C}$ as constructed above. Since the model $\mathcal{C}$ is convex, the classical trial model in the general case is still $\mathcal{C}$.

B. Construction of PEFs

We observe that the classical model $\mathcal{C}$ obtained above is convex, so we need only to ensure that the PEF inequality in Eq. (1) of the main text is satisfied at each extremal distribution of $\mathcal{C}$ according to Lemma 14 of Ref. [1]. However, the set $\mathcal{C}$ has an infinite number of extremal distributions, and so a PEF needs to satisfy an infinite number of linear constraints, where each extremal distribution of $\mathcal{C}$ imposes a linear constraint. In order to effectively construct PEFs by numerical optimization, instead we can find a convex polytope $\mathcal{P}$ that has a finite number of extreme points and contains the classical model $\mathcal{C}$ of interest. In this way, if a non-negative function $F_{\mathrm{c}} : (i, o) \mapsto F_{\mathrm{c}}(i, o)$ satisfies all the PEF inequalities imposed by the finite number of extreme points of $\mathcal{P}$, then the function $F_{\mathrm{c}} : (i, o) \mapsto F_{\mathrm{c}}(i, o)$ will also

satisfy all the PEF inequalities imposed by each distribution in $\mathcal{C}$ (see Lemma 14 of Ref. [1]). Therefore, the function $F_{\rm c} : (i, o) \mapsto F_{\rm c}(i, o)$ is a PEF for the classical model $\mathcal{C}$. Below we will first explain how to find such a convex polytope $\mathcal{P}$ and then present the explicit optimization problem for constructing PEFs.

For the classical model $\mathcal{C}$ constructed according to Eq. (7), each member $\mathbb{P}(I, O)$ of $\mathcal{C}$ is given by the product of a member $\mathbb{P}(I)$ in a closed interval $\mathcal{S}$ and a member $\mathbb{P}(O|I)$ in the convex set $\mathcal{C}_{O|I}$. Hence, in order to find a convex polytope $\mathcal{P}$ such that $\mathcal{C} \subseteq \mathcal{P}$, it suffices to find another convex polytope $\mathcal{P}_{O|I}$ such that $\mathcal{C}_{O|I} \subseteq \mathcal{P}_{O|I}$. To achieve this goal, in view of the construction of $\mathcal{C}_{O|I}$ given in Eq. (6) we just need to find a convex polygon $\mathcal{R}'$ such that $\mathcal{R} \subseteq \mathcal{R}'$. Since $\mathcal{R} = q_{1,\rm lb}\mathcal{R}_1 + (1 - q_{1,\rm lb})\mathcal{R}_s$ (see Eq. (4)) and the region $\mathcal{R}_s$ is a square, we need only to find a convex polygon $\mathcal{R}_1'$ such that $\mathcal{R}_1 \subseteq \mathcal{R}_1'$. Since the boundary of the region $\mathcal{R}_1$ is well characterized (see Eq. (3) and Supplementary Figure 1), we can find such a convex polygon $\mathcal{R}_1'$ as detailed in the next paragraph.

We choose $\mathcal{R}_1'$ to be a convex polygon circumscribed around $\mathcal{R}_1$. For this, we observe that in each quadrant the boundary of $\mathcal{R}_1$ is composed by two line segments and one elliptic curve. For example, in the first quadrant the two line segments are shown in the first and third lines of Eq. (3) and the curve is shown in the second line of Eq. (3), that is, $\langle\sigma_X\rangle + \langle\sigma_Z\rangle = \sqrt{2 + 2\sin(\delta)}\cos(\psi)$ and $\langle\sigma_X\rangle - \langle\sigma_Z\rangle = \sqrt{2 - 2\sin(\delta)}\sin(\psi)$, where $\psi \in (-\phi, \phi)$ and $\phi = \arcsin(\sqrt{(1 - \sin(\delta))/2})$. Consequently, if we choose $(2K - 1)$ points in the elliptic curve of each quadrant, for example, in the first quadrant the points characterized by $\psi = 0, \pm\phi/K, \pm 2\phi/K, ..., \pm(K - 1)\phi/K$, and draw tangent lines of the curve at these points, then the region $\mathcal{R}_1'$ bounded by these tangent lines and the line segments in the boundary of $\mathcal{R}_1$ is a $(8K)$-sided convex polygon circumscribed around $\mathcal{R}_1$. Therefore, following the arguments of the previous paragraph, we can obtain a convex polytope $\mathcal{P}$ circumscribed around the convex set $\mathcal{C}$. Moreover, with the increase of the value for K, the resulting convex polytopes $\mathcal{R}_1'$ and $\mathcal{P}$ become better outer-approximations of their respective convex regions $\mathcal{R}_1$ and $\mathcal{C}$. In our numerical construction of PEFs detailed below, we choose $K = 64$.

Now we are ready to present the optimization problem for constructing PEFs. We emphasize that to ensure the validity of the subsequent security analysis using PEFs, a PEF for a trial needs to be constructed before preforming the trial. Since the classical model for each trial is the identical $\mathcal{C}$, we can use the same PEF, $F_{\rm c} : (i, o) \mapsto F_{\rm c}(i, o)$, for each trial, and construct this PEF before the experiment. With the same PEF for each trial, from Theorem 1 of the main text we know that if

$$\sum_{k=1}^{n} \log_2(F_{\rm c}(I_k, O_k))/\beta_{\rm c} + \log_2(\epsilon_s)/\beta_{\rm c} \geq -\log_2(p), \tag{9}$$

the classical ϵ_s-smooth conditional min-entropy certified in a successful experiment with n trials (without taking into account the probability of success) is at least $-\log_2(p)$. Therefore, the quantity in the left-hand side of Eq. (9) can be informally interpreted as the amount of classical ϵ_s-smooth min-entropy in the outputs $\mathbf{O}_n$ certifiable conditionally on the inputs $\mathbf{I}_n$ and the classical side information E. Hence, before the experiment we need to choose a PEF such that the expected value of the quantity in the left-hand side of Eq. (9) is as large as possible. For this purpose, we temporarily assume that the experimental trials are independent and identically distributed (i.i.d.). Suppose that the actual distribution of each trial's input and output is $\mathbb{P}_{\rm exp}(I, O)$ and that the classical trial model $\mathcal{C}$ is contained in a convex polytope $\mathcal{P}$ with L extremal distributions $\mathbb{P}_l(I, O)$, $l = 1, 2, ..., L$. Then, to construct a well-performing PEF with a proper power $\beta_{\rm c}$, before the experiment we can solve the following optimization problem:

$$\begin{array}{ll} \text{Maximize} & \sum_{i,o} n\mathbb{P}_{\rm exp}(I = i, O = o)\log_2(F_{\rm c}(i, o))/\beta_{\rm c} + \log_2(\epsilon_s)/\beta_{\rm c} \\ \text{Subject to:} & 1)\ \sum_{i,o} \mathbb{P}_l(I = i, O = o)F_{\rm c}(i, o)\mathbb{P}_l(O = o|I = i)^{\beta_{\rm c}} \leq 1, l = 1, 2, ..., L, \\ & 2)\ F_{\rm c}(i, o) \geq 0, \forall i, o. \end{array} \tag{10}$$

The above maximization is over both the power $\beta_{\rm c} > 0$ and the PEF $F_{\rm c} : (i, o) \mapsto F_{\rm c}(i, o)$. When fixing the power $\beta_{\rm c}$, the objective function is a strictly concave function of the PEF $F_{\rm c}$ and the constraints are linear in $F_{\rm c}$, so there is a unique maximum. This maximum and the corresponding PEF can be found by the sequential quadratic programming [1, 2]. After solving the maximization over the PEFs with a fixed power $\beta_{\rm c}$, the maximization over $\beta_{\rm c}$ can be solved by a local search. We would like to emphasize that as long as a function $F_{\rm c} : (i, o) \mapsto F_{\rm c}(i, o)$ satisfies the constraints in the optimization problem of Eq. (10), the function is a valid PEF with power $\beta_{\rm c}$ for the classical trial model $\mathcal{C}$. That is, the validity of the constructed PEF depends only on the validity of the classical model $\mathcal{C}$. This has two implications: First, the PEF constructed by the above maximization is valid no matter what the actual distribution $\mathbb{P}_{\rm exp}(I, O)$ of the trial's input and output is. Hence, in practice it is not necessary to learn the actual distribution $\mathbb{P}_{\rm exp}(I, O)$ before the experiment, and in the optimization problem of Eq. (10) we can replace $\mathbb{P}_{\rm exp}(I, O)$ by a distribution $\mathbb{P}_{\rm est}(I, O)$ estimated based on the calibration data that is available before the experiment (see Supplementary Note 4 for details). Second, even if the trials are not i.i.d. (but are characterized by the same classical model $\mathcal{C}$), the constructed PEF is still valid.

Finally we define the quantity

$$R_{\mathrm{c}} = \max_{\beta_{\mathrm{c}}>0} \max_{F_{\mathrm{c}}} \Big(\sum_{i,o} \mathbb{P}_{\mathrm{exp}}(I=i, O=o) \log_2(F_{\mathrm{c}}(i,o))/\beta_{\mathrm{c}} \Big). \tag{11}$$

Here the optimization is over both the power $\beta_{\mathrm{c}} > 0$ and the PEF $F_{\mathrm{c}} : (i,o) \mapsto F_{\mathrm{c}}(i,o)$ for the classical trial model $\mathcal{C}$. In view of the arguments in the previous paragraph, R_{c} can be identified as the asymptotic randomness-generation rate per trial witnessed by all possible PEFs when each trial has the same distribution $\mathbb{P}_{\mathrm{exp}}(I,O)$ and is characterized by the same model $\mathcal{C}$, see Refs. [1, 2] for detailed justifications. Moreover, the asymptotic rate R_{c} is optimal, see Sect. VI D of Ref. [2] for a general proof. We can bound the asymptotic rate R_{c} from below by constructing a convex polytope $\mathcal{P}$ with L extremal distributions such that $\mathcal{P} \supseteq \mathcal{C}$. Specifically, using the same numerical method as the above for constructing PEFs we can find a lower bound on R_{c}. With the increase of the value for L, we obtain a tighter lower bound on R_{c}. To obtain the results presented in Figure 1 of the main text, we chose $L = 1024$. This choice is justified by our observation that the obtained lower bound on R_{c} would not increase further if a larger value for L is used.

Supplementary Note 2. Quantum probability estimation for our experiment

In this supplementary note, we present the details of how to implement the framework of quantum probability estimation for certifying randomness against quantum side information [3, 4]. We note that for device-independent randomness generation, the excellent finite-data efficiency of quantum probability estimation has been demonstrated in Refs. [3–5]. Here we would like to apply quantum probability estimation for low-latency randomness generation with partially characterized quantum devices. In Supplementary Note 2 A and Supplementary Note 2 B, we construct the quantum model for each trial and the corresponding quantum estimation factors (QEFs) for the experiment of our interest, respectively. At each trial, the input distribution $\mathbb{P}(I)$ is not exactly known but is chosen from the convex set $\mathcal{S}$ of Eq. (5). We emphasize that quantum probability estimation is a general framework for certifying randomness against quantum side information; however, the implementation of this framework is case-dependent.

In this supplementary note, we identify classical systems with random variables. We denote quantum systems by upper-case letters in sans serif font (such as D, E). Throughout this supplementary note, E plays a distinguished role as the system carrying the quantum side information. We denote the identity operator on a quantum system by $\mathbb{1}$. Quantum states, unless otherwise specified, refer to normalized quantum states.

A. Construction of the quantum model

To construct the quantum model for a trial, we first temporarily assume that Eve's quantum side information about the state is independent of Eve's partial knowledge of the input and measurement at the trial. Then we remark and explain that the quantum model in the general case where Eve's quantum side information about the state is classically correlated with Eve's partial knowledge of the input and measurement can be easily obtained as the convex closure of the model $\mathcal{Q}$ constructed with the above independence assumption. The model $\mathcal{Q}$ can be constructed by considering all the possible combinations of Eve's partial knowledge of the input and measurement (i.e., all the possible input distributions and all the possible measurements on the quantum system D of the device used in our experiment) and Eve's quantum side information about the state (i.e., all the possible quantum states ρ_{DE} shared between the device's system D and Eve's system E before the trial). The construction of $\mathcal{Q}$ is detailed in the following two steps:

Step 1. *We consider the case where at each trial the photon source emits a single photon with probability* 1. In this case, the device's system D_1 is described as a qubit, and the two measurements on the device are described as $\sigma_X = \boldsymbol{\sigma} \cdot \mathbf{n}_X$ and $\sigma_Z = \boldsymbol{\sigma} \cdot \mathbf{n}_Z$ where $|\mathbf{n}_X \cdot \mathbf{n}_Z| \leq \sin(\delta)$. Each measurement is a projection-valued measure (PVM) on the system D_1. Let $\Pi_{\mathsf{D}_1,o|i}$ be the input-dependent PVM element for the trial output $O = o$ given the trial input $I = i$, where $i = X$ or Z and $o = +1$ or -1. Suppose that before the trial, the joint state of the systems D_1 and E is $\rho_{\mathsf{D}_1\mathsf{E}}$. Given a trial input i, the state of the trial output O and Eve's system E is induced by performing the corresponding measurement on the initial state $\rho_{\mathsf{D}_1\mathsf{E}}$. That is,

$$\rho_{O\mathsf{E}|i} = \sum_o |o\rangle\langle o| \otimes \mathrm{Tr}_{\mathsf{D}_1} \big(\rho_{\mathsf{D}_1\mathsf{E}} (\Pi_{\mathsf{D}_1,o|i} \otimes \mathbb{1}_{\mathsf{E}}) \big), \tag{12}$$

where $\mathrm{Tr}_{\mathsf{D}_1}$ is the partial trace over the system D_1 and $\mathbb{1}_{\mathsf{E}}$ is the identity operator on the system E. If we assume that the input I at each trial can be arbitrarily chosen with probability distribution $\mathbb{P}(I)$ in the convex set $\mathcal{S}$ of Eq. (5),

then the joint state of the trial input I, the trial output O and Eve's system E is a classical-quantum state of the form

$$\rho_{IO\mathsf{E}} = \sum_i \mathbb{P}(I=i)|i\rangle\langle i| \otimes \rho_{O\mathsf{E}|i}, \tag{13}$$

where $\mathbb{P}(I) \in \mathcal{S}$. Since the initial state $\rho_{\mathsf{D}_1\mathsf{E}}$ of the systems D_1 and E is arbitrary, the measurement directions satisfy $|\mathbf{n}_X \cdot \mathbf{n}_Z| \le \sin(\delta)$, and the input distributions $\mathbb{P}(I)$ is arbitrarily chosen from the convex set $\mathcal{S}$, we obtain a set of classical-quantum states $\rho_{IO\mathsf{E}}$. This set is the quantum model $\mathcal{Q}_1$ for each trial under the assumption that the device's system is a qubit. Note that the quantum model $\mathcal{Q}_1$ in general is not convex but has a convex closure $\bar{\mathcal{Q}}_1$. To construct QEFs for the trial model $\mathcal{Q}_1$, considering Property 2 of Ref. [3] we need only to characterize the extremal states in the convex set $\bar{\mathcal{Q}}_1$ and the corresponding set of sandwiched Rényi powers, which are detailed in the next two paragraphs respectively.

It is obvious to see that the initial state $\rho_{\mathsf{D}_1\mathsf{E}}$ should be a pure state in order to ensure the resulting state $\rho_{IO\mathsf{E}}$ is extremal in $\bar{\mathcal{Q}}_1$. Since the system D_1 is a qubit, by the Schmidt decomposition a pure state $|\psi\rangle_{\mathsf{D}_1\mathsf{E}}$ can be written as $|\psi\rangle_{\mathsf{D}_1\mathsf{E}} = \cos(\mu)|\bar{0}\rangle_{\mathsf{D}_1}|\bar{0}\rangle_{\mathsf{E}} + \sin(\mu)|\bar{1}\rangle_{\mathsf{D}_1}|\bar{1}\rangle_{\mathsf{E}}$ in the Schmidt basis $\{\bar{0}, \bar{1}\}$ with $\mu \in [0, \pi/2]$. Suppose that the two measurement directions $\mathbf{n}_X$ and $\mathbf{n}_Z$ are in the X-Z plane of the Bloch sphere, and let $|0\rangle$ and $|1\rangle$ be the two eigenstates of the measurement operator $\sigma_Z = \boldsymbol{\sigma} \cdot \mathbf{n}_Z$ with eigenvalues $+1$ and -1 respectively. Then, in the basis $\{|0\rangle, |1\rangle\}$ the two measurements σ_X and σ_Z, as well as their corresponding PVM elements, are represented by real matrices. In general, the Schmidt basis states $|\bar{0}\rangle$ and $|\bar{1}\rangle$ can be not in the X-Z plane of the Bloch sphere, that is, the density matrix $\rho_{\mathsf{D}_1\mathsf{E}} = |\psi\rangle_{\mathsf{D}_1\mathsf{E}}\langle\psi|$ can be not a real matrix in the basis $\{|0\rangle, |1\rangle\}$. However, since all PVM elements $\Pi_{\mathsf{D}_1,o|i}$ with $i = X, Z$ and $o = \pm 1$ are represented by real matrices in the basis $\{|0\rangle, |1\rangle\}$, the density matrix $\rho_{\mathsf{D}_1\mathsf{E}}$ must be also represented by a real matrix in the same basis in order to ensure the resulting state $\rho_{IO\mathsf{E}}$ is extremal. Therefore, the Schmidt basis states $|\bar{0}\rangle$ and $|\bar{1}\rangle$ should be in the X-Z plane of the Bloch sphere. In other words, to obtain the extremal states of the convex closure $\bar{\mathcal{Q}}_1$ of the quantum model $\mathcal{Q}_1$, we need only to consider pure initial states $|\psi\rangle_{\mathsf{D}_1\mathsf{E}}$ with the Schmidt basis states being in the X-Z plane of the Bloch sphere. In particular, suppose that the Schmidt basis states are $|\bar{0}\rangle = \cos(\gamma)|0\rangle + \sin(\gamma)|1\rangle$ and $|\bar{1}\rangle = \sin(\gamma)|0\rangle - \cos(\gamma)|1\rangle$, where $\gamma \in [0, \pi)$, and let $\mathbf{n}_X = (\sin(\theta), -\cos(\theta))$ and $\mathbf{n}_Z = (0, 1)$ be two unit vectors in the X-Z plane of the Bloch sphere, where $\theta \in [\pi/2 - \delta, \pi/2 + \delta]$. Then, the extremal states of the convex closure $\bar{\mathcal{Q}}_1$ of the quantum model $\mathcal{Q}_1$ can be expressed as

$$\rho_{IO\mathsf{E}} = \sum_{i,o} |i,o\rangle\langle i,o| \otimes \rho_{\mathsf{E}}(i,o), \tag{14}$$

where the sub-normalized states $\rho_{\mathsf{E}}(i,o)$ of E conditional on $I = i$ and $O = o$ are represented in the Schmidt basis $\{|\bar{0}\rangle, |\bar{1}\rangle\}$ as follows:

$$\begin{aligned}
\rho_{\mathsf{E}}(I=X, O=+1) &= \mathbb{P}(I=X) \begin{bmatrix} \cos^2(\mu)\sin^2(\gamma+\theta/2) & -\sin(2\mu)\sin(2\gamma+\theta)/4 \\ -\sin(2\mu)\sin(2\gamma+\theta)/4 & \sin^2(\mu)\cos^2(\gamma+\theta/2) \end{bmatrix}, \\
\rho_{\mathsf{E}}(I=X, O=-1) &= \mathbb{P}(I=X) \begin{bmatrix} \cos^2(\mu)\cos^2(\gamma+\theta/2) & \sin(2\mu)\sin(2\gamma+\theta)/4 \\ \sin(2\mu)\sin(2\gamma+\theta)/4 & \sin^2(\mu)\sin^2(\gamma+\theta/2) \end{bmatrix}, \\
\rho_{\mathsf{E}}(I=Z, O=+1) &= \mathbb{P}(I=Z) \begin{bmatrix} \cos^2(\gamma)\cos^2(\mu) & \sin(2\gamma)\sin(2\mu)/4 \\ \sin(2\gamma)\sin(2\mu)/4 & \sin^2(\gamma)\sin^2(\mu) \end{bmatrix}, \\
\rho_{\mathsf{E}}(I=Z, O=-1) &= \mathbb{P}(I=Z) \begin{bmatrix} \sin^2(\gamma)\cos^2(\mu) & -\sin(2\gamma)\sin(2\mu)/4 \\ -\sin(2\gamma)\sin(2\mu)/4 & \cos^2(\gamma)\sin^2(\mu) \end{bmatrix}.
\end{aligned} \tag{15}$$

Now we are ready to characterize the set $\mathcal{R}_{\bar{\mathcal{Q}}_1,e}$ of sandwiched Rényi powers according to the extremal states of the convex set $\bar{\mathcal{Q}}_1$. As the PVM elements $\Pi_{\mathsf{D}_1,o|i}$ for all i and o are rank-1 and the initial state $\rho_{\mathsf{D}_1\mathsf{E}}$ is pure, we observe that the sub-normalized states $\rho_{\mathsf{E}}(i,o)$ in Eq. (15) are rank-1. With this observation in mind and in view of

the definition of sandwiched Rényi powers in Eq. (4) of the main text, direct calculation shows that

$$\begin{aligned}
R(\rho_{\mathsf{E}}, X, +1) &= \mathbb{P}(I=X)\left(\sin^2(\gamma+\theta/2)\cos^{2/\alpha_{\rm q}}(\mu)+\cos^2(\gamma+\theta/2)\sin^{2/\alpha_{\rm q}}(\mu)\right)^{\alpha_{\rm q}},\\
R(\rho_{\mathsf{E}}, X, -1) &= \mathbb{P}(I=X)\left(\cos^2(\gamma+\theta/2)\cos^{2/\alpha_{\rm q}}(\mu)+\sin^2(\gamma+\theta/2)\sin^{2/\alpha_{\rm q}}(\mu)\right)^{\alpha_{\rm q}},\\
R(\rho_{\mathsf{E}}, Z, +1) &= \mathbb{P}(I=Z)\left(\cos^2(\gamma)\cos^{2/\alpha_{\rm q}}(\mu)+\sin^2(\gamma)\sin^{2/\alpha_{\rm q}}(\mu)\right)^{\alpha_{\rm q}},\\
R(\rho_{\mathsf{E}}, Z, -1) &= \mathbb{P}(I=Z)\left(\sin^2(\gamma)\cos^{2/\alpha_{\rm q}}(\mu)+\cos^2(\gamma)\sin^{2/\alpha_{\rm q}}(\mu)\right)^{\alpha_{\rm q}},
\end{aligned} \tag{16}$$

where we abbreviate the sandwiched Rényi power $R_{\alpha_{\rm q}}\big(\rho_{\mathsf{E}}(I=i,O=o)\big|\rho_{\mathsf{E}}(I=i)\big)$ as $R(\rho_{\mathsf{E}},i,o)$ for each $i=X$ or Z and $o=+1$ or -1. Denote the vector $\big(R(\rho_{\mathsf{E}},X,+1),R(\rho_{\mathsf{E}},X,-1),R(\rho_{\mathsf{E}},Z,+1),R(\rho_{\mathsf{E}},Z,-1)\big)$ by $\mathbf{R}(\rho_{\mathsf{E}})$. Then, the set $\mathcal{R}_{\bar{\mathcal{Q}}_1,e}$ is given as

$$\begin{aligned}
\mathcal{R}_{\bar{\mathcal{Q}}_1,e} = \{&\mathbf{R}(\rho_{\mathsf{E}}), \text{ where } R(\rho_{\mathsf{E}},i,o) \text{ with } i{=}X \text{ or } Z \text{ and } o=+1 \text{ or } -1, \text{ each entry of } \mathbf{R}(\rho_{\mathsf{E}}),\\
&\text{has the form of Eq. (16) with } \mu\in[0,\pi/2],\ \gamma\in[0,\pi) \text{ and } \theta\in[\pi/2-\delta,\pi/2+\delta]\}.
\end{aligned} \tag{17}$$

We have two remarks on the constructions of the quantum model $\mathcal{Q}_1$ and the corresponding QEFs as follows. First, in view of the convexity of $\bar{\mathcal{Q}}_1 \supseteq \mathcal{Q}_1$ and the joint convexity of sandwiched Rényi powers (Proposition 3 of Ref. [6]), the convex closure of the set $\mathcal{R}_{\bar{\mathcal{Q}}_1,e}$, which is denoted by $\bar{\mathcal{R}}_{\bar{\mathcal{Q}}_1,e}$, satisfies the following property: Each element in the set $\mathcal{R}_{\mathcal{Q}_1}$ of sandwiched Rényi powers according to all the states in the model $\mathcal{Q}_1$ is bounded from above by an element in the set $\bar{\mathcal{R}}_{\bar{\mathcal{Q}}_1,e}$. We denote the above relation by $\mathcal{R}_{\mathcal{Q}_1} \leq \bar{\mathcal{R}}_{\bar{\mathcal{Q}}_1,e}$. Therefore, if a non-negative function $F_{\rm q}:(i,o)\mapsto F_{\rm q}(i,o)$ satisfies the QEF inequalities imposed by all the elements of the set $\bar{\mathcal{R}}_{\bar{\mathcal{Q}}_1,e}$, then this function will also satisfy the QEF inequalities imposed by all the elements of the set $\mathcal{R}_{\mathcal{Q}_1}$. In other words, if a function $F_{\rm q}:(i,o)\mapsto F_{\rm q}(i,o)$ is a QEF with respect to the sandwiched Rényi powers in the set $\bar{\mathcal{R}}_{\bar{\mathcal{Q}}_1,e}$, then this function is a QEF with respect to the sandwiched Rényi powers in the set $\mathcal{R}_{\mathcal{Q}_1}$ according to the quantum model $\mathcal{Q}_1$. This observation applies to a general quantum model. In this work, we will construct the QEFs for the quantum model $\mathcal{Q}$ of our interest by characterizing the set of sandwiched Rényi powers according to the convex closure of $\mathcal{Q}$. Second, suppose that the two measurement directions $\mathbf{n}_X$ and $\mathbf{n}_Z$ are in the X-Z plane of the Bloch sphere. Without loss of generality, let $\mathbf{n}_X=(\cos(\theta_X),\sin(\theta_X))$ and $\mathbf{n}_Z=(\sin(\theta_Z),\cos(\theta_Z))$, where $|\theta_X|\leq\delta_X$, $|\theta_Z|\leq\delta_Z$ and $\delta_X+\delta_Z\leq\delta$ satisfying the bound on the misalignment. Denote the PVM element for the trial output o given the trial input i by $\Pi^{\theta_X}_{\mathsf{D}_1,o|i=X}$ or $\Pi^{\theta_Z}_{\mathsf{D}_1,o|i=Z}$, where the dependence on the measurement direction is made explicit. Then, given arbitrary $|\theta_X|\leq\delta_X$ and $|\theta_Z|\leq\delta_Z$, an arbitrary initial state $\rho_{\mathsf{D}_1\mathsf{E}}$, and an input distribution $\mathbb{P}(I)$ in the convex set $\mathcal{S}$ of Eq. (5), the resulting classical-quantum state $\rho^{(\theta_X,\theta_Z)}_{IO\mathsf{E}}$ is in the quantum model $\mathcal{Q}_1$. Now consider the two POVMs

$$M_X=\Big\{\sum_k \omega_{X,k}\Pi^{\theta_{X,k}}_{\mathsf{D}_1,o=+1|i=X},\sum_k \omega_{X,k}\Pi^{\theta_{X,k}}_{\mathsf{D}_1,o=-1|i=X}\Big\} \text{ and } M_Z=\Big\{\sum_l \omega_{Z,l}\Pi^{\theta_{Z,l}}_{\mathsf{D}_1,o=+1|i=Z},\sum_l \omega_{Z,l}\Pi^{\theta_{Z,l}}_{\mathsf{D}_1,o=-1|i=Z}\Big\}, \tag{18}$$

where for all k (l), $\omega_{X,k}\geq 0$ and $|\theta_{X,k}|\leq\delta_X$ ($\omega_{Z,l}\geq 0$ and $|\theta_{Z,l}|\leq\delta_Z$), and $\sum_k\omega_{X,k}=\sum_l\omega_{Z,l}=1$. The classical-quantum state induced by performing the above two POVMs selected randomly at a trial can be expressed as a convex mixture

$$\rho_{IO\mathsf{E}}=\sum_{k,l}\omega_{X,k}\omega_{Z,l}\rho^{(\theta_{X,k},\theta_{Z,l})}_{IO\mathsf{E}}, \tag{19}$$

where each state $\rho^{(\theta_{X,k},\theta_{Z,l})}_{IO\mathsf{E}}$ is induced by performing the assumed projective measurements selected randomly at a trial and thus in the quantum model $\mathcal{Q}_1$. So, the classical-quantum state $\rho_{IO\mathsf{E}}$ in Eq. (19) is in the convex closure $\bar{\mathcal{Q}}_1$ of the quantum model $\mathcal{Q}_1$. Hence, the quantum model induced by the above POVMs is generally larger than the quantum model $\mathcal{Q}_1$ induced by the assumed projective measurements, but both models have the same convex closure. In view of the first remark above and the remarks below Eq. (5) of the main text, a QEF works well for both a quantum model and its convex closure. Therefore, in our construction of the quantum model not only the assumed projective measurements but also the POVMs of the form in Eq. (18) are allowed. We emphasize that the set of POVMs considered above does not include all the possible POVMs that are close to the ideal projective measurement σ_1 or σ_3. Also, our current method does not allow Eve to hold purifications of POVMs, as it assumes that Eve can access classical side information but *not* quantum side information about the measurement performed at a trial. How to completely handle general POVMs with our method deserves further investigation in future work.

Step 2. *We consider the general case where at each trial the probability of emitting a single photon from the photon*

source is at least $q_{1,lb}$. In view of the fact that the measurements on the quantum system D of the device are block-diagonal with respect to various photon-number subspaces, without loss of generality the states ρ_{DE} shared between the device's system D and Eve's system E before the trial can be restricted to those with the same block-diagonal structure. That is, ρ_{DE} has the direct-sum (denoted by $\oplus$) structure

$$\rho_{\mathsf{DE}} = \rho_{\mathsf{D}_1\mathsf{E}} \oplus \rho_{\mathsf{D}_2\mathsf{E}}, \tag{20}$$

where D_1 is a qubit system corresponding to the case that the photon source emits a single photon, and D_2 is a quantum system of arbitrary dimension corresponding to the case that the photon source emits multiple photons or zero photon. Moreover, the sub-normalized state $\rho_{\mathsf{D}_1\mathsf{E}}$ satisfies the constraint that $\mathrm{Tr}(\rho_{\mathsf{D}_1\mathsf{E}}) \geq q_{1,\mathrm{lb}}$ in order to be consistent with the single-photon probability bound allowed. For the model construction, we conservatively assume that both the state of the subsystem D_2 and the measurements on D_2 are completely uncharacterized except that each measurement has two outcomes ± 1. As the dimension of the system D_2 can be arbitrarily high, the measurements on D_2 can be restricted to projective ones. Let $\Pi_{\mathsf{D}_2,o|i}$ be the input-dependent PVM element on D_2 for the trial output $O = o$ given the trial input $I = i$. Recall that the input-dependent PVM elements on D_1 are denoted by $\Pi_{\mathsf{D}_1,o|i}$ and that they are partially characterized. Suppose that before the trial, the joint state of the systems D and E is ρ_{DE} of the form in Eq. (20). Then, given a trial input i, the state of the trial output O and Eve's system E induced by performing the corresponding measurement is

$$\rho_{O\mathsf{E}|i} = \rho^{(1)}_{O\mathsf{E}|i} \bigoplus \rho^{(2)}_{O\mathsf{E}|i}, \tag{21}$$

where $\rho^{(k)}_{O\mathsf{E}|i} = \sum_o |o\rangle\langle o| \otimes \mathrm{Tr}_{\mathsf{D}_k}\left(\rho_{\mathsf{D}_k\mathsf{E}}(\Pi_{\mathsf{D}_k,o|i} \otimes \mathbb{1}_{\mathsf{E}})\right)$, $k = 1, 2$. Suppose that the input distribution $\mathbb{P}(I)$ is arbitrarily chosen from the convex set $\mathcal{S}$ of Eq. (5). The joint state of the trial input I, the trial output O and Eve's system E is a classical-quantum state of the form

$$\rho_{IO\mathsf{E}} = \sum_i \mathbb{P}(I = i)|i\rangle\langle i| \otimes \rho_{O\mathsf{E}|i} = \rho^{(1)}_{IO\mathsf{E}} \bigoplus \rho^{(2)}_{IO\mathsf{E}}, \tag{22}$$

where $\rho^{(k)}_{IO\mathsf{E}} = \sum_i \mathbb{P}(I = i)|i\rangle\langle i| \otimes \rho^{(k)}_{O\mathsf{E}|i}$, $k = 1, 2$, and $\mathbb{P}(I) \in \mathcal{S}$.

To construct the quantum model, let us introduce the normalized states $\tilde{\rho}^{(k)}_{IO\mathsf{E}} = \rho^{(k)}_{IO\mathsf{E}}/\mathrm{Tr}(\rho_{\mathsf{D}_k\mathsf{E}})$, $k = 1, 2$. Since the joint of the systems D and E has an arbitrary initial state ρ_{DE} of the form in Eq. (20), all the possible states $\tilde{\rho}^{(1)}_{IO\mathsf{E}}$ achievable at a trial form the set $\mathcal{Q}_1$ studied in Step 1. Denote the set of all possible states $\tilde{\rho}^{(2)}_{IO\mathsf{E}}$ by $\mathcal{Q}_2$ and its convex closure by $\bar{\mathcal{Q}}_2$. We observe that $\mathcal{Q}_2$ is convex and so $\mathcal{Q}_2 = \bar{\mathcal{Q}}_2$, considering the following facts: 1) both the sub-normalized state $\rho_{\mathsf{D}_2\mathsf{E}}$ and the measurements on the system D_2 are arbitrary; 2) the input distribution $\mathbb{P}(I)$ is arbitrarily chosen from the convex set $\mathcal{S}$ of Eq. (5). When the trace $\mathrm{Tr}(\rho_{\mathsf{D}_1\mathsf{E}})$ is fixed to a value t, all the possible states $\rho_{IO\mathsf{E}}$ achievable at a trial form the set

$$\mathcal{Q}^t = \{\rho_{IO\mathsf{E}} = t\tilde{\rho}^{(1)}_{IO\mathsf{E}} \bigoplus (1-t)\tilde{\rho}^{(2)}_{IO\mathsf{E}}, \text{ where } \tilde{\rho}^{(1)}_{IO\mathsf{E}} \in \mathcal{Q}_1 \text{ and } \tilde{\rho}^{(2)}_{IO\mathsf{E}} \in \mathcal{Q}_2\}. \tag{23}$$

In the case of our interest, the trace $\mathrm{Tr}(\rho_{\mathsf{D}_1\mathsf{E}})$ takes a value at least $q_{1,\mathrm{lb}}$, so the set of classical-quantum states $\rho_{IO\mathsf{E}}$ achievable at a trial is the union of all the sets $\mathcal{Q}^t$ where $t \geq q_{1,\mathrm{lb}}$. This union is the quantum model $\mathcal{Q}$ for each trial assuming that the single-photon probability is bounded from below by $q_{1,\mathrm{lb}}$. Specifically, the quantum model $\mathcal{Q}$ can be expressed as

$$\mathcal{Q} = \{\rho_{IO\mathsf{E}} = t\tilde{\rho}^{(1)}_{IO\mathsf{E}} \bigoplus (1-t)\tilde{\rho}^{(2)}_{IO\mathsf{E}}, \text{ where } \tilde{\rho}^{(1)}_{IO\mathsf{E}} \in \mathcal{Q}_1,\ \tilde{\rho}^{(2)}_{IO\mathsf{E}} \in \mathcal{Q}_2 \text{ and } t \geq q_{1,\mathrm{lb}}\}. \tag{24}$$

Note that the set $\mathcal{Q}$ in general is not convex. We also remark that Eq. (24) implies the statement in the main text that the quantum model $\mathcal{Q}$ can be expressed as a direct sum of sub-models constructed conditionally on a varying number of photons emitted from the source. To construct QEFs for the trial model $\mathcal{Q}$, we need to characterize the set $\mathcal{R}_{\mathcal{Q}}$ of sandwiched Rényi powers according to all the states in $\mathcal{Q}$, which is detailed in the next three paragraphs.

We first exploit the block-diagonal structure of the classical-quantum states in the quantum model $\mathcal{Q}$ to simplify the characterization of $\mathcal{R}_{\mathcal{Q}}$. For all $\rho_{IO\mathsf{E}} \in \mathcal{Q}$ and for all trial inputs i and trial outputs o, we have that

$$\rho_{\mathsf{E}}(i,o) = \rho^{(1)}_{\mathsf{E}}(i,o) \bigoplus \rho^{(2)}_{\mathsf{E}}(i,o) \text{ and } \rho_{\mathsf{E}}(i) = \rho^{(1)}_{\mathsf{E}}(i) \bigoplus \rho^{(2)}_{\mathsf{E}}(i), \tag{25}$$

where $\rho^{(k)}_{\mathsf{E}}(i,o) = \mathbb{P}(I = i)\,\mathrm{Tr}_{\mathsf{D}_k}\left(\rho_{\mathsf{D}_k\mathsf{E}}(\Pi_{\mathsf{D}_k,o|i} \otimes \mathbb{1}_{\mathsf{E}})\right)$ and $\rho^{(k)}_{\mathsf{E}}(i) = \sum_o \rho^{(k)}_{\mathsf{E}}(i,o)$, $k = 1, 2$. That is, $\rho_{\mathsf{E}}(i,o)$ and $\rho_{\mathsf{E}}(i)$

for all inputs i and outputs o are simultaneously block-diagonal. Considering this fact and in view of the definition in Eq. (4) of the main text, direct calculation shows that the sandwiched Rényi powers satisfy

$$\begin{aligned} R_{\alpha_{\rm q}}\big(\rho_{\mathsf{E}}(i,o)\big|\rho_{\mathsf{E}}(i)\big) &= R_{\alpha_{\rm q}}\big(\rho^{(1)}_{\mathsf{E}}(i,o)\big|\rho^{(1)}_{\mathsf{E}}(i)\big) + R_{\alpha_{\rm q}}\big(\rho^{(2)}_{\mathsf{E}}(i,o)\big|\rho^{(2)}_{\mathsf{E}}(i)\big) \\ &= \mathrm{Tr}(\rho_{\mathsf{D}_1\mathsf{E}})R_{\alpha_{\rm q}}\big(\tilde{\rho}^{(1)}_{\mathsf{E}}(i,o)\big|\tilde{\rho}^{(1)}_{\mathsf{E}}(i)\big) + \mathrm{Tr}(\rho_{\mathsf{D}_2\mathsf{E}})R_{\alpha_{\rm q}}\big(\tilde{\rho}^{(2)}_{\mathsf{E}}(i,o)\big|\tilde{\rho}^{(2)}_{\mathsf{E}}(i)\big), \end{aligned} \tag{26}$$

where $\tilde{\rho}^{(k)}_{\mathsf{E}}(i,o)=\rho^{(k)}_{\mathsf{E}}(i,o)/\mathrm{Tr}(\rho_{\mathsf{D}_k\mathsf{E}})$ and $\tilde{\rho}^{(k)}_{\mathsf{E}}(i)=\rho^{(k)}_{\mathsf{E}}(i)/\mathrm{Tr}(\rho_{\mathsf{D}_k\mathsf{E}})$, $k=1,2$. In other words, a sandwiched Rényi power according to the normalized state $\rho_{IO\mathsf{E}}=\rho^{(1)}_{IO\mathsf{E}}\bigoplus\rho^{(2)}_{IO\mathsf{E}}$ is given by the probabilistic mixture of sandwiched Rényi powers according to the normalized states $\tilde{\rho}^{(k)}_{IO\mathsf{E}}=\rho^{(k)}_{IO\mathsf{E}}/\mathrm{Tr}(\rho_{\mathsf{D}_k\mathsf{E}})$, $k=1,2$. Since $\rho_{IO\mathsf{E}}$ is an arbitrary state in the quantum model $\mathcal{Q}$ and $\tilde{\rho}^{(k)}_{IO\mathsf{E}}$ is the corresponding state in the set $\mathcal{Q}_k$, $k=1,2$, any sandwiched Rényi power according to $\mathcal{Q}$ can be written as a probabilistic mixture of sandwiched Rényi powers according to $\mathcal{Q}_k$, $k=1,2$. Specifically, let the sets of sandwiched Rényi powers according to $\mathcal{Q}_1$ and $\mathcal{Q}_2$ be $\mathcal{R}_{\mathcal{Q}_1}$ and $\mathcal{R}_{\mathcal{Q}_2}$ respectively. Then we have

$$\mathcal{R}_{\mathcal{Q}}=\bigcup_{1\geq\omega\geq q_{1,\mathrm{lb}}}\big(\omega\mathcal{R}_{\mathcal{Q}_1}+(1-\omega)\mathcal{R}_{\mathcal{Q}_2}\big), \tag{27}$$

considering that $\omega=\mathrm{Tr}(\rho_{\mathsf{D}_1\mathsf{E}})\geq q_{1,\mathrm{lb}}$. Recall that we already characterized the set $\mathcal{R}_{\mathcal{Q}_1}$ in Step 1. Precisely speaking, we characterized a convex set $\bar{\mathcal{R}}_{\bar{\mathcal{Q}}_1,e}$ such that $\mathcal{R}_{\mathcal{Q}_1}\leq\bar{\mathcal{R}}_{\bar{\mathcal{Q}}_1,e}$, see the paragraph below Eq. (17).

Second, we characterize the set $\mathcal{R}_{\mathcal{Q}_2}$. In particular, to construct QEFs we need only to characterize the set $\mathcal{R}_{\bar{\mathcal{Q}}_2,e}$ of sandwiched Rényi powers according to the extremal states of the convex set $\mathcal{Q}_2=\bar{\mathcal{Q}}_2$. So, we need to first characterize the extremal states of $\bar{\mathcal{Q}}_2$. Since there are only two binary-outcome measurements on the device, by the lemma of Ref. [7] or Lemma 2 of Ref. [8] there exists an orthonormal basis for the system D_2 where the PVM elements of the two measurements are simultaneously block-diagonal with blocks of size at most 2×2. Therefore, the system D_2 can be assumed to be a qubit in order to obtain the extremal states of $\bar{\mathcal{Q}}_2$. So we reduce to the case considered in Step 1. The only difference lies in that the two projective measurements on D_2 are conservatively assumed to be completely uncharacterized such that the angle θ between the two measurement directions on the Bloch sphere can be arbitrary. Hence, the extremal states of $\bar{\mathcal{Q}}_2$ have the form of Eq. (14), where the sub-normalized states $\rho_{\mathsf{E}}(i,o)$ are given in Eq. (15) with the parameters $\mu\in[0,\pi/2]$, $\gamma\in[0,\pi)$ and $\theta\in[0,\pi]$. According to these extremal states, Eve's probability of correctly guessing the output O given the input I at a trial can be as large as 1, meaning that no randomness can be certified according to the quantum model $\mathcal{Q}_2=\bar{\mathcal{Q}}_2$. Recall that the quantum model $\mathcal{Q}_2$ is constructed under the conservative assumption that both the state of the subsystem D_2 and the measurements on D_2 are completely uncharacterized. We thus like to remark that with the above assumption, we choose to not certify the randomness contributed by the outcomes obtained from measurements on the subsystem D_2, and so our security analysis is robust against photon-number splitting attacks. Now we can characterize the set $\mathcal{R}_{\bar{\mathcal{Q}}_2,e}$. Similar to the set $\mathcal{R}_{\bar{\mathcal{Q}}_1,e}$ in Eq. (17), the set $\mathcal{R}_{\bar{\mathcal{Q}}_2,e}$ can be expressed as

$$\begin{aligned} \mathcal{R}_{\bar{\mathcal{Q}}_2,e}=\{&\mathbf{R}(\rho_{\mathsf{E}}),\text{ where } R(\rho_{\mathsf{E}},i,o)\text{ with } i{=}X\text{ or }Z\text{ and } o=+1\text{ or }-1,\text{ each entry of }\mathbf{R}(\rho_{\mathsf{E}}),\\ &\text{has the form of Eq. (16) with }\mu\in[0,\pi/2],\ \gamma\in[0,\pi)\text{ and }\theta\in[0,\pi]\}. \end{aligned} \tag{28}$$

In view of the convexity of $\bar{\mathcal{Q}}_2$ and the joint convexity of sandwiched Rényi powers (Proposition 3 of Ref. [6]), the convex closure of the set $\mathcal{R}_{\bar{\mathcal{Q}}_2,e}$, which is denoted by $\bar{\mathcal{R}}_{\bar{\mathcal{Q}}_2,e}$, satisfies the following property: Each element in the set $\mathcal{R}_{\mathcal{Q}_2}$ is bounded from above by an element in the set $\bar{\mathcal{R}}_{\bar{\mathcal{Q}}_2,e}$. We denote the above relation by $\mathcal{R}_{\mathcal{Q}_2}\leq\bar{\mathcal{R}}_{\bar{\mathcal{Q}}_2,e}$. In the same sense, below we extend the bound relation "$\leq$" to two arbitrary sets.

Third and now we are ready to characterize the set $\mathcal{R}_{\mathcal{Q}}$. In view of the results in the above two paragraphs, we have $\mathcal{R}_{\mathcal{Q}_k}\leq\bar{\mathcal{R}}_{\bar{\mathcal{Q}}_k,e}$, $k=1,2$, and so

$$\big(\omega\mathcal{R}_{\mathcal{Q}_1}+(1-\omega)\mathcal{R}_{\mathcal{Q}_2}\big)\leq\big(\omega\bar{\mathcal{R}}_{\bar{\mathcal{Q}}_1,e}+(1-\omega)\bar{\mathcal{R}}_{\bar{\mathcal{Q}}_2,e}\big),\forall\omega\in[0,1]. \tag{29}$$

Here, for a fixed ω the set $\big(\omega\bar{\mathcal{R}}_{\bar{\mathcal{Q}}_1,e}+(1-\omega)\bar{\mathcal{R}}_{\bar{\mathcal{Q}}_2,e}\big)$ is convex as both sets $\bar{\mathcal{R}}_{\bar{\mathcal{Q}}_1,e}$ and $\bar{\mathcal{R}}_{\bar{\mathcal{Q}}_2,e}$ are convex. By Eqs. (17) and (28), we also have that $\mathcal{R}_{\bar{\mathcal{Q}}_1,e}\subseteq\mathcal{R}_{\bar{\mathcal{Q}}_2,e}$ and so $\bar{\mathcal{R}}_{\bar{\mathcal{Q}}_1,e}\subseteq\bar{\mathcal{R}}_{\bar{\mathcal{Q}}_2,e}$. Therefore, we get

$$\big(\omega\bar{\mathcal{R}}_{\bar{\mathcal{Q}}_1,e}+(1-\omega)\bar{\mathcal{R}}_{\bar{\mathcal{Q}}_2,e}\big)\subseteq\big(q_{1,\mathrm{lb}}\bar{\mathcal{R}}_{\bar{\mathcal{Q}}_1,e}+(1-q_{1,\mathrm{lb}})\bar{\mathcal{R}}_{\bar{\mathcal{Q}}_2,e}\big),\forall\omega\in[q_{1,\mathrm{lb}},1]. \tag{30}$$

In view of Eqs. (27), (29) and (30), we thus get the relation

$$\mathcal{R}_{\mathcal{Q}} \leq \bar{\mathcal{R}}_{\bar{\mathcal{Q}}}, \tag{31}$$

where $\bar{\mathcal{R}}_{\bar{\mathcal{Q}}} = \left(q_{1,\text{lb}}\mathcal{R}_{\bar{\mathcal{Q}}_1,e} + (1-q_{1,\text{lb}})\mathcal{R}_{\bar{\mathcal{Q}}_2,e}\right)$ is a convex set. The above relation is useful for constructing QEFs, as detailed in Supplementary Note 2 B.

We emphasize that to construct the quantum model $\mathcal{Q}$, we exploit only the fact that the measurements M_X and M_Z are block-diagonal with respect to various photon-number subspaces, and we do not use any information about the normalized state in each photon-number subspace. The measurement operators and the distribution of photon numbers emitted from the source, as well as the input distribution, may depend on some auxiliary degrees of freedom, such as spatial mode, which are not used for encoding information. If Eve can access these auxiliary degrees of freedom such as by a side-channel attack, Eve could be able to manipulate the measurements performed and the quantum state prepared. In this case, we take advantage of the fact that in practice the measurements are block-diagonal with respect to various states for the auxiliary degrees of freedom manipulable by Eve (see the Methods section of the main text for detailed explanations). Therefore, as long as the adversarial imperfections are characterized by the parameters $(q_{1,\text{lb}}, \delta, \tau_{\text{lb}}, \tau_{\text{ub}})$, the quantum model $\mathcal{Q}$ constructed as above, as well as the QEFs constructed below, is valid, meaning that our security analysis is robust against such side-channel attacks.

We finally remark that in the general case where Eve's quantum side information about the state is classically correlated with Eve's partial knowledge of the input and measurement at a trial, the joint state of the trial input I, the trial output O and Eve's system E can be written as

$$\rho_{IO\mathsf{E}} = \sum_{\lambda} \omega_{\Lambda=\lambda}\rho_{IO\mathsf{E}}^{\Lambda=\lambda}, \tag{32}$$

where for each λ, $\omega_{\Lambda=\lambda} \geq 0$, and $\sum_\lambda \omega_{\Lambda=\lambda} = 1$. Here, the random variable Λ characterizes the classical correlation between Eve's quantum side information about the state and Eve's partial knowledge of the input and measurement, and for each possible value $\Lambda = \lambda$, the classical-quantum state $\rho_{IO\mathsf{E}}^{\Lambda=\lambda}$ is in the model $\mathcal{Q}$ of Eq. (24). Therefore, the quantum model for the trial will become the convex closure $\bar{\mathcal{Q}}$ of the model $\mathcal{Q}$ as characterized above. In view of the remarks below Eq. (5) of the main text, no matter whether the quantum model for a trial is $\mathcal{Q}$ or $\bar{\mathcal{Q}}$ the set of QEFs constructed is the same. For this reason, without loss of generality the quantum model for a trial is characterized by the model $\mathcal{Q}$ of Eq. (24). But we emphasize that the construction of QEFs detailed in the next section and so our security analysis with respect to quantum side information work in the general case where Eve's quantum side information about the state is classically correlated with Eve's partial knowledge of the input and measurement at a trial.

B. Construction of QEFs

Considering the relation in Eq. (31) and the joint convexity of sandwiched Rényi powers (Proposition 3 of Ref. [6]), to construct QEFs for the quantum model $\mathcal{Q}$ it suffices to consider the QEF inequalities imposed by the extreme points of the convex set $\bar{\mathcal{R}}_{\bar{\mathcal{Q}}}$. These extreme points are expressed as

$$\mathbf{R}_{\bar{\mathcal{Q}},e} = q_{1,\text{lb}}\mathbf{R}_{\bar{\mathcal{Q}}_1,e} + (1-q_{1,\text{lb}})\mathbf{R}_{\bar{\mathcal{Q}}_2,e}, \tag{33}$$

where $\mathbf{R}_{\bar{\mathcal{Q}}_1,e}$ and $\mathbf{R}_{\bar{\mathcal{Q}}_2,e}$ are in the sets $\mathcal{R}_{\bar{\mathcal{Q}}_1,e}$ and $\mathcal{R}_{\bar{\mathcal{Q}}_2,e}$ of Eq. (17) and Eq. (28) respectively. However, the set $\bar{\mathcal{R}}_{\bar{\mathcal{Q}}}$ has an infinite number of extreme points, and so a QEF needs to satisfy an infinite number of linear constraints, where each extreme point of $\bar{\mathcal{R}}_{\bar{\mathcal{Q}}}$ imposes a linear constraint. In order to effectively construct QEFs by numerical optimization, instead we can find a convex polytope $\mathcal{P}$ that has a finite number of extreme points and contains the convex set $\bar{\mathcal{R}}_{\bar{\mathcal{Q}}}$. In this way, if a non-negative function $F_{\text{q}} : (i,o) \mapsto F_{\text{q}}(i,o)$ satisfies all the QEF inequalities imposed by the finite number of extreme points of $\mathcal{P}$, then the function $F_{\text{q}} : (i,o) \mapsto F_{\text{q}}(i,o)$ will also satisfy all the QEF inequalities imposed by each member of the convex set $\bar{\mathcal{R}}_{\bar{\mathcal{Q}}}$ (see Property 2 of Ref. [3]). Therefore, the function $F_{\text{q}} : (i,o) \mapsto F_{\text{q}}(i,o)$ is a QEF for the quantum model $\mathcal{Q}$. Below we will first explain how to find such a convex polytope $\mathcal{P}$ and then present the explicit optimization problem for constructing QEFs.

Since the extreme points of $\bar{\mathcal{R}}_{\bar{\mathcal{Q}}}$ can be expressed as in Eq. (33), to find a convex polytope $\mathcal{P}$ such that $\bar{\mathcal{R}}_{\bar{\mathcal{Q}}} \subseteq \mathcal{P}$ we need only to find another two polytopes $\mathcal{P}_1$ and $\mathcal{P}_2$ such that $\mathcal{R}_{\bar{\mathcal{Q}}_1,e} \subseteq \mathcal{P}_1$ and $\mathcal{R}_{\bar{\mathcal{Q}}_2,e} \subseteq \mathcal{P}_2$. Because the elements $\mathbf{R}(\rho_{\mathsf{E}})$ of the two sets $\mathcal{R}_{\bar{\mathcal{Q}}_1,e}$ (Eq. (17)) and $\mathcal{R}_{\bar{\mathcal{Q}}_2,e}$ (Eq. (28)) are expressed as the same function of parameters γ, θ and μ (they are different only in the range of the parameter θ), we can find the two polytopes $\mathcal{P}_1$ and $\mathcal{P}_2$ in the same way. Our strategy for finding $\mathcal{P}_1$ and $\mathcal{P}_2$ is outlined as follows: We express the vector $\mathbf{R}(\rho_{\mathsf{E}})$ as the entry-wise product of another two vectors, and then find out two convex polytopes which contain the accessible regions of these

two vectors respectively. Consequently, all the accessible vectors $\mathbf{R}(\rho_{\mathsf{E}})$ are contained in a polytope whose extreme points are given by the entry-wise products of the extreme points of the two polytopes found above. The details of our strategy are presented in the next two paragraphs.

In view of Eq. (16), we first write $\mathbf{R}(\rho_{\mathsf{E}})$ as the entry-wise product of two vectors $\mathbf{p}$ and $\mathbf{q}(\rho_{\mathsf{E}})$, where the entries of $\mathbf{p}$ and $\mathbf{q}(\rho_{\mathsf{E}})$ are denoted as $p(i,o)$ and $q(\rho_{\mathsf{E}},i,o)$ respectively. We choose $p(i,o)=\mathbb{P}(I=i)$ and $q(\rho_{\mathsf{E}},i,o)=r(\rho_{\mathsf{E}},i,o)^{\alpha_{\mathrm{q}}}$, where each $r(\rho_{\mathsf{E}},i,o)$ is non-negative and given by the inner product (denoted by $\cdot$) of two vectors as follows:

$$\begin{aligned}
r(\rho_{\mathsf{E}},X,+1)&=\big(\sin^2(\gamma+\theta/2),\cos^2(\gamma+\theta/2)\big)\cdot\big(\cos^{2/\alpha_{\mathrm{q}}}(\mu),\sin^{2/\alpha_{\mathrm{q}}}(\mu)\big),\\
r(\rho_{\mathsf{E}},X,-1)&=\big(\cos^2(\gamma+\theta/2),\sin^2(\gamma+\theta/2)\big)\cdot\big(\cos^{2/\alpha_{\mathrm{q}}}(\mu),\sin^{2/\alpha_{\mathrm{q}}}(\mu)\big),\\
r(\rho_{\mathsf{E}},Z,+1)&=\big(\cos^2(\gamma),\sin^2(\gamma)\big)\cdot\big(\cos^{2/\alpha_{\mathrm{q}}}(\mu),\sin^{2/\alpha_{\mathrm{q}}}(\mu)\big),\\
r(\rho_{\mathsf{E}},Z,-1)&=\big(\sin^2(\gamma),\cos^2(\gamma)\big)\cdot\big(\cos^{2/\alpha_{\mathrm{q}}}(\mu),\sin^{2/\alpha_{\mathrm{q}}}(\mu)\big).
\end{aligned}\tag{34}$$

Recall that the input distribution $\mathbb{P}(I)$ is arbitrarily chosen from a closed interval $\mathcal{S}$ of Eq. (5), which is a convex polytope. So, secondly we need to find a convex polytope that contains all the accessible vectors $\mathbf{q}(\rho_{\mathsf{E}})$. As $q(\rho_{\mathsf{E}},i,o)=r(\rho_{\mathsf{E}},i,o)^{\alpha_{\mathrm{q}}}$ with $\alpha_{\mathrm{q}}>1$ is a convex function of $r(\rho_{\mathsf{E}},i,o)$ and $r(\rho_{\mathsf{E}},i,o)$ is non-negative, it suffices to find a convex polytope that contains all the accessible vectors $\mathbf{r}(\rho_{\mathsf{E}})\doteq(r(\rho_{\mathsf{E}},X,+1),r(\rho_{\mathsf{E}},X,-1),r(\rho_{\mathsf{E}},Z,+1),r(\rho_{\mathsf{E}},Z,-1))$. To see this, suppose that $\mathbf{r}(\rho_{\mathsf{E}})=\sum_g\omega_g\mathbf{r}_g$ with $\omega_g\geq 0$ and $\sum_g\omega_g=1$. Then $\mathbf{q}(\rho_{\mathsf{E}})\leq\sum_g\omega_g\mathbf{q}_g$, where the entries of $\mathbf{q}_g$ are given by the α_{q}-th powers of the entries of $\mathbf{r}_g$. As $\mathbf{p}$ is a vector with non-negative entries, if a non-negative function $F_{\mathrm{q}}:(i,o)\mapsto F_{\mathrm{q}}(i,o)$ satisfies all the QEF inequalities imposed by $\mathbf{p}\cdot\mathbf{q}_g$, then the function $F_{\mathrm{q}}:(i,o)\mapsto F_{\mathrm{q}}(i,o)$ satisfies the QEF inequality imposed by $\mathbf{p}\cdot\mathbf{q}(\rho_{\mathsf{E}})$. Therefore, the convex polytope with extreme points $\mathbf{q}_g$ suffices for our purpose of constructing QEFs. To find a convex polytope containing all accessible $\mathbf{r}(\rho_{\mathsf{E}})$, we introduce the vectors $\mathbf{v}_1=(1/2,1/2)$, $\mathbf{v}_2=(1/2,-1/2)$ and $\mathbf{v}_\mu=\big(\cos^{2/\alpha_{\mathrm{q}}}(\mu),\sin^{2/\alpha_{\mathrm{q}}}(\mu)\big)$. Then, the expressions in Eq. (34) can be rewritten as

$$\begin{aligned}
r(\rho_{\mathsf{E}},X,+1)&=(\mathbf{v}_1-\cos(2\gamma+\theta)\mathbf{v}_2)\cdot\mathbf{v}_\mu,\\
r(\rho_{\mathsf{E}},X,-1)&=(\mathbf{v}_1+\cos(2\gamma+\theta)\mathbf{v}_2)\cdot\mathbf{v}_\mu,\\
r(\rho_{\mathsf{E}},Z,+1)&=(\mathbf{v}_1+\cos(2\gamma)\mathbf{v}_2)\cdot\mathbf{v}_\mu,\\
r(\rho_{\mathsf{E}},Z,-1)&=(\mathbf{v}_1-\cos(2\gamma)\mathbf{v}_2)\cdot\mathbf{v}_\mu.
\end{aligned}\tag{35}$$

Let us introduce another vector $\mathbf{v}_{\gamma,\theta}=\big(\cos(2\gamma+\theta),\cos(2\gamma)\big)$. As detailed in the next paragraph, for all values of γ, θ and μ we can find convex decompositions

$$\mathbf{v}_{\gamma,\theta}=\sum_k\omega_{k,\gamma,\theta}(a_k,b_k)\text{ and }\mathbf{v}_\mu=\sum_m\omega_{m,\mu}(c_m,d_m),\tag{36}$$

where $\omega_{k,\gamma,\theta},\omega_{m,\mu}\geq 0$, $\sum_k\omega_{k,\gamma,\theta}=\sum_m\omega_{m,\mu}=1$, and all a_k,b_k,c_m,d_m are constants independent of γ, θ and μ. Then from Eqs. (35) and (36) we obtain that

$$\begin{aligned}
r(\rho_{\mathsf{E}},X,+1)&=\sum_{k,m}\omega_{k,\gamma,\theta}\omega_{m,\mu}(\mathbf{v}_1-a_k\mathbf{v}_2)\cdot(c_m,d_m)=\sum_{k,m}\omega_{k,\gamma,\theta}\omega_{m,\mu}\big((1-a_k)c_m/2+(1+a_k)d_m/2\big),\\
r(\rho_{\mathsf{E}},X,-1)&=\sum_{k,m}\omega_{k,\gamma,\theta}\omega_{m,\mu}(\mathbf{v}_1+a_k\mathbf{v}_2)\cdot(c_m,d_m)=\sum_{k,m}\omega_{k,\gamma,\theta}\omega_{m,\mu}\big((1+a_k)c_m/2+(1-a_k)d_m/2\big),\\
r(\rho_{\mathsf{E}},Z,+1)&=\sum_{k,m}\omega_{k,\gamma,\theta}\omega_{m,\mu}(\mathbf{v}_1+b_k\mathbf{v}_2)\cdot(c_m,d_m)=\sum_{k,m}\omega_{k,\gamma,\theta}\omega_{m,\mu}\big((1+b_k)c_m/2+(1-b_k)d_m/2\big),\\
r(\rho_{\mathsf{E}},Z,-1)&=\sum_{k,m}\omega_{k,\gamma,\theta}\omega_{m,\mu}(\mathbf{v}_1-b_k\mathbf{v}_2)\cdot(c_m,d_m)=\sum_{k,m}\omega_{k,\gamma,\theta}\omega_{m,\mu}\big((1-b_k)c_m/2+(1+b_k)d_m/2\big).
\end{aligned}\tag{37}$$

Denote the vector $\big((1-a_k)c_m/2+(1+a_k)d_m/2,(1+a_k)c_m/2+(1-a_k)d_m/2,(1+b_k)c_m/2+(1-b_k)d_m/2,(1-b_k)c_m/2+(1+b_k)d_m/2\big)$ by $\mathbf{r}_{km}$. Eq. (37) tells us that all accessible vectors $\mathbf{r}(\rho_{\mathsf{E}})$ can be written as convex combinations of the vectors $\mathbf{r}_{km}$. Therefore, the convex polytope defined as the convex closure of the points $\mathbf{r}_{km}$ contains all the accessible vectors $\mathbf{r}(\rho_{\mathsf{E}})$.

In this paragraph we find the convex decompositions in Eq. (36). We first consider the set of vectors $\mathbf{v}_{\gamma,\theta}=\big(\cos(2\gamma+\theta),\cos(2\gamma)\big)$. To obtain the elements $\mathbf{R}(\rho_{\mathsf{E}})$ in both sets $\mathcal{R}_{\bar{\mathcal{Q}}_1,e}$ (Eq. (17)) and $\mathcal{R}_{\bar{\mathcal{Q}}_2,e}$ (Eq. (28)), $\gamma\in[0,\pi)$. So, the set of vectors $\mathbf{v}_{\gamma,\theta}$ is the same as the set of vectors $\mathbf{v}'_{\gamma,\theta}=\big(\cos(2\gamma+\theta/2),\cos(2\gamma-\theta/2)\big)$. Let $\langle\sigma_X\rangle=\cos(2\gamma+\theta/2)$

and $\langle\sigma_Z\rangle = \cos(2\gamma - \theta/2)$. Then the set of vectors $\mathbf{v}_{\gamma,\theta}$ ($\mathbf{v}'_{\gamma,\theta}$) is described by the ellipse

$$\frac{(\langle\sigma_X\rangle + \langle\sigma_Z\rangle)^2}{2+2\cos(\theta)} + \frac{(\langle\sigma_X\rangle - \langle\sigma_Z\rangle)^2}{2-2\cos(\theta)} = 1, \tag{38}$$

which corresponds to Eq. (2) for constructing the classical trial model. For the elements $\mathbf{R}(\rho_{\mathsf{E}})$ in the set $\mathcal{R}_{\bar{\mathcal{Q}}_1,e}$ of Eq. (17) (or in the set $\mathcal{R}_{\bar{\mathcal{Q}}_2,e}$ of Eq. (28)), the parameter θ takes values in the interval $[\pi/2-\delta, \pi/2+\delta]$ (or in the interval $[0,\pi]$, corresponding to the case $\delta = \pi/2$). Hence, the set of vectors $\mathbf{v}_{\gamma,\theta}$ is contained in the convex set $\mathcal{R}_1$ specified below Eq. (2). We note that the shape of the convex set $\mathcal{R}_1$ depends on the value of δ. In Supplementary Note 1 B we already found a convex polygon $\mathcal{R}'_1$ such that $\mathcal{R}_1 \subseteq \mathcal{R}'_1$. So, we can use the extreme points of $\mathcal{R}'_1$ as the vectors (a_k, b_k) for the convex decompositions of the vectors $\mathbf{v}_{\gamma,\theta}$. Specifically, for the vectors $\mathbf{v}_{\gamma,\theta}$ used for characterizing the set $\mathcal{R}_{\bar{\mathcal{Q}}_1,e}$ where $\theta \in [\pi/2-\delta, \pi/2+\delta]$, we choose $\mathcal{R}'_1$ be a convex polygon with $K_1 = 512$ extreme points, while for the vectors $\mathbf{v}_{\gamma,\theta}$ used for characterizing the set $\mathcal{R}_{\bar{\mathcal{Q}}_2,e}$ where $\theta \in [0,\pi]$ (that is, $\delta = \pi/2$), $\mathcal{R}_1$ becomes $\mathcal{R}_s$, a square with 4 extreme points $(1,1), (1,-1), (-1,1)$ and $(-1,-1)$, and so we choose $\mathcal{R}'_1$ to be $\mathcal{R}_s$. Secondly, we consider the set of vectors $\mathbf{v}_\mu = \left(\cos^{2/\alpha_{\mathrm{q}}}(\mu), \sin^{2/\alpha_{\mathrm{q}}}(\mu)\right)$. To obtain the elements $\mathbf{R}(\rho_{\mathsf{E}})$ in both sets $\mathcal{R}_{\bar{\mathcal{Q}}_1,e}$ (Eq. (17)) and $\mathcal{R}_{\bar{\mathcal{Q}}_2,e}$ (Eq. (28)), $\mu \in [0, \pi/2]$. So, the set of vectors $\mathbf{v}_\mu$ is represented by a curve S_μ in the first quadrant starting at the point $(1,0)$ and ending at the point $(0,1)$. To obtain a convex polygon which contains the curve S_μ, we choose (K_2-1) points in the curve S_μ, for example, the points characterized by $\mu = \phi/(2K_2), 2\phi/(2K_2), ..., (K_2-1)\phi/(2K_2)$, and draw tangent lines of the curve S_μ at these points. The region bounded by these tangent lines and the line connecting the two points $(1,0)$ and $(0,1)$ is a convex polygon circumscribing around the curve S_μ. Therefore, with the extreme points of this convex polygon, we can express all the vectors $\mathbf{v}_\mu$ by convex decompositions. In our numerical construction of QEFs detailed below, we choose $K_2 = 64$. We note that with the increase of the values for K_1 and K_2, the resulting convex polygons become better outer-approximations of the regions accessible by the vectors $\mathbf{v}_{\gamma,\theta}$ and $\mathbf{v}_\mu$ respectively.

Now we are ready to present the optimization problem for constructing QEFs. We emphasize that to ensure the validity of the subsequent security analysis using QEFs, a QEF for a trial needs to be constructed before preforming the trial. Since the quantum model for each trial is the identical $\mathcal{Q}$, we can use the same QEF, $F_{\mathrm{q}} : (i,o) \mapsto F_{\mathrm{q}}(i,o)$, for each trial, and construct this QEF before the experiment. With the same QEF for each trial, from Theorem 2 of the main text we know that if

$$\sum_{k=1}^{n} \log_2(F_{\mathrm{q}}(I_k, O_k))/\beta_{\mathrm{q}} + \log_2(\epsilon_s^2/2)/\beta_{\mathrm{q}} \geq -\log_2(p), \tag{39}$$

the quantum ϵ_s-smooth conditional min-entropy certified in a successful experiment with n trials (without taking into account the probability of success) is at least $-\log_2(p)$. Therefore, the quantity in the left-hand side of Eq. (39) can be informally interpreted as the amount of quantum ϵ_s-smooth min-entropy in the outputs $\mathbf{O}_n$ certifiable conditionally on the inputs $\mathbf{I}_n$ and the quantum side information in E. Hence, before the experiment we need to choose a QEF such that the expected value of the quantity in the left-hand side of Eq. (39) is as large as possible. For this purpose, we temporarily assume that the experimental trials are i.i.d. Suppose that the actual distribution of each trial's input and output is $\mathbb{P}_{\mathrm{exp}}(I,O)$ and that the set $\mathcal{R}_{\mathcal{Q}}$ of sandwiched Rényi powers according to the quantum model $\mathcal{Q}$ is contained in a convex polytope $\mathcal{P}$ with L extreme points $\mathbf{R}_l$, $l = 1, 2, ..., L$, where the entries of $\mathbf{R}_l$ are $R_l(i,o)$ for different inputs $i = X$ or Z and outputs $o = +1$ or -1. We note that the set $\mathcal{R}_{\mathcal{Q}}$ and the polytope $\mathcal{P}$ as well as the extreme points of $\mathcal{P}$ all depend implicitly on the power parameter β_{q} and the parameters ($q_{1,\mathrm{lb}}$, δ, τ_{lb}, τ_{ub}) which characterize the adversarial imperfections in the experiment. To construct a well-performing QEF with a proper power β_{q}, before the experiment we can solve the following optimization problem:

$$\begin{array}{ll} \text{Maximize} & \sum_{i,o} n\mathbb{P}_{\mathrm{exp}}(I=i, O=o)\log_2(F_{\mathrm{q}}(i,o))/\beta_{\mathrm{q}} + \log_2(\epsilon_s^2/2)/\beta_{\mathrm{q}} \\ \text{Subject to:} & 1)\ \sum_{i,o} F_{\mathrm{q}}(i,o) R_l(i,o) \leq 1, l = 1, 2, ..., L, \\ & 2)\ F_{\mathrm{q}}(i,o) \geq 0, \forall i, o. \end{array} \tag{40}$$

The above maximization is over both the power $\beta_{\mathrm{q}} > 0$ and the QEF $F_{\mathrm{q}} : (i,o) \mapsto F_{\mathrm{q}}(i,o)$. When fixing the power β_{q}, the objective function is a strictly concave function of the QEF F_{q} and the constraints are linear in F_{q}, so there is a unique maximum. This maximum and the corresponding QEF can be found by the sequential quadratic programming, the same algorithm as that for constructing PEFs. After solving the maximization over the QEFs with a fixed power β_{q}, the maximization over β_{q} can be solved by a local search. We would like to emphasize that as long as a function $F_{\mathrm{q}} : (i,o) \mapsto F_{\mathrm{q}}(i,o)$ satisfies the constraints in the optimization problem of Eq. (40), the function is a valid QEF with power β_{q} for the quantum trial model $\mathcal{Q}$. That is, the validity of the constructed QEF depends only on the validity of the quantum model $\mathcal{Q}$. This has two implications: First, the QEF constructed by the above maximization is valid no

matter what the actual distribution $\mathbb{P}_{\exp}(I,O)$ of the trial's input and output is. Hence, in practice it is not necessary to learn the actual distribution $\mathbb{P}_{\exp}(I,O)$ before the experiment, and in the optimization problem of Eq. (40) we can replace $\mathbb{P}_{\exp}(I,O)$ by a distribution $\mathbb{P}_{\rm est}(I,O)$ estimated based on the calibration data that is available before the experiment (see Supplementary Note 4 for details). Second, even if the trials are not i.i.d. (but are characterized by the same quantum model $\mathcal{Q}$), the constructed QEF is still valid.

Finally we define the quantity

$$R_{\rm q} = \max_{\beta_{\rm q}>0} \max_{F_{\rm q}} \Big(\sum_{i,o} \mathbb{P}_{\exp}(I=i, O=o) \log_2(F_{\rm q}(i,o))/\beta_{\rm q} \Big). \tag{41}$$

Here the optimization is over both the power $\beta_{\rm q} > 0$ and the QEF $F_{\rm q} : (i,o) \mapsto F_{\rm q}(i,o)$ for the quantum trial model $\mathcal{Q}$. In view of the arguments in the previous paragraph, $R_{\rm q}$ can be identified as the asymptotic randomness-generation rate per trial witnessed by all possible QEFs when each trial has the same distribution $\mathbb{P}_{\exp}(I,O)$ and is characterized by the same model $\mathcal{Q}$, see Refs. [3, 4] for detailed justifications. Moreover, the asymptotic rate $R_{\rm q}$ is optimal, see Sect. 6.5 of Ref. [4] for a general proof. We can bound the asymptotic rate $R_{\rm q}$ from below by constructing a convex polytope $\mathcal{P}$ with L extreme points such that $\mathcal{P} \supseteq \mathcal{R}_{\mathcal{Q}}$. Specifically, using the same numerical method as the above for constructing QEFs we can find a lower bound on $R_{\rm q}$. With the increase of the value for L, we obtain a tighter lower bound on $R_{\rm q}$. To obtain the results presented in Figure 1 of the main text, we chose $L = 675840$. This choice is justified by our observation that the obtained lower bound on $R_{\rm q}$ would not increase further if a larger value for L is used.

Supplementary Note 3. Imperfections in our experiment

The imperfections to be characterized depend on the specific implementation of the QRNG scheme and can be bounded by calibrating the photon source and measurement apparatus in real time. The lower bound $q_{1,\rm lb}$ on the single-photon probability for constructing the models and performing the corresponding security analyses can be obtained by monitoring the average photon number per trial given the photon-number distribution. The bounds $\tau_{\rm lb}$ and $\tau_{\rm ub}$ on the imbalance $\tau = (P_X - P_Z)/2$ depend on how the measurement basis at a trial is selected. For the active-selection scheme, a random number from some pseudo- or physical randomness source is used and so a calibration of this randomness source is needed. For the passive-selection scheme like in our experiment, beam splitters are used and so a calibration of their splitting ratios is needed. The calibration of the misalignment angle δ also depends on the setup. For the active-selection scheme with polarization encoding, the measurement directions are set by a polarization rotator driven by some electric or magnetic field. Because of the fluctuation of the electric or magnetic field in practice, the measurement directions set by the rotator can vary from trial to trial. Therefore, we need to calibrate the electric or magnetic field applied. For the passive-selection scheme with time-bin encoding as in our experiment, the measurement directions are determined by the splitting ratios of the two beam splitters in the interferometer used as well as by the efficiency mismatch of the two detectors employed (see our characterization detailed below). Therefore, we need to calibrate the splitting ratios and the efficiency mismatch. The imperfections in the measurement apparatus of our experiment are characterized in Supplementary Note 3 A, while the imperfections in our photon source are in Supplementary Note 3 B.

A. Imperfections in measurement apparatus

We perform measurements on photonic time-bin states with an unbalanced Mach-Zehnder interferometer (MZI) as shown in Supplementary Figure 2 (1). The MZI has one input spatial mode, which can transmit two different temporal modes—the early and late time-bin modes labelled as e and l respectively, and has two output spatial modes labelled as a and b respectively. The difference in photon transit time between the two unbalanced paths of the MZI matches the separation between the two time-bin modes e and l. Therefore, depending on which path an input photon in the early (late) time-bin mode takes, the photon can arrive at the output of the MZI in the early or middle time-bin mode (in the middle or late time-bin mode). So, the output photon can be in three different temporal modes, the early, middle and late time-bin modes labelled as e, m and l respectively. Considering both the temporal and spatial modes, the MZI is described by an optical device with two input modes and six output modes as shown in Supplementary Figure 2 (2). We remark that for detecting photons at the two output spatial modes, threshold detectors (which cannot resolve photon number) of finite efficiency are employed. Below we derive the measurement operators in the single-photon subspace and characterize their dependence on imperfections considering the following two cases: 1) The two detectors employed have the same detection efficiency, and 2) the two detectors at the output ports a and

b have efficiencies η_a and η_b respectively. We emphasize that for constructing the classical and quantum models and performing the corresponding security analyses, our method requires a characterization of the measurement operators in the single-photon subspace but not those operators in the multiphoton subspace.

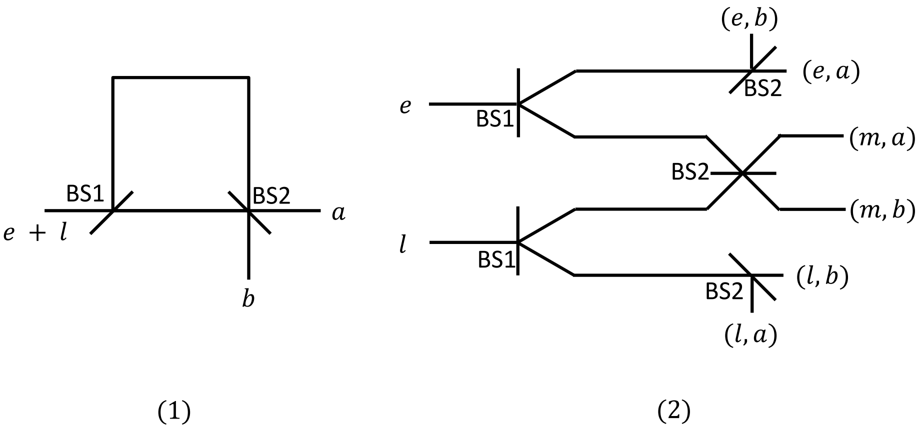

Supplementary Figure 2: Two equivalent descriptions of an unbalanced Mach-Zehnder interferometer (MZI). The left panel (1) is the description in the experiment. The MZI is composed of two beam splitters, BS1 and BS2, and has two input temporal modes (the early and late time-bin modes labelled as e and l) and two output spatial modes (labelled as a and b). The right panel (2) is the hypothetical description which helps to illustrate the relations between the input and output modes. Here we separate different combinations of the spatial and temporal modes for the input and output of the MZI. Note that the output can be in three different temporal modes, the early, middle and late time-bin modes (labelled as e, m and l).

Case 1. We assume that the detectors employed are trusted such that their detection efficiencies are the same and also independent of any degree of freedom of the incoming photons. As a consequence, the no-click events due to detection inefficiency can be treated as an attenuation of the incoming optical pulse before performing measurements with threshold detectors of perfect efficiency. (The effect of optical-pulse attenuations will be discussed in Supplementary Note 3 B.) So, without loss of generality we assume that each of the detectors employed has the perfect efficiency.

In the ideal case, each of the two beam splitters BS1 and BS2 in the MZI has the 50:50 splitting ratio. However, in practice it is difficult to ensure the ideal splitting ratio. Suppose that the two beam splitters BS1 and BS2 have transmission coefficients t_1 and t_2 respectively. The coefficients t_1 and t_2 may depend on the auxiliary degrees of freedom of the single photon which are not used for information encoding but manipulable by Eve. As explained in the Methods section of the main text, we take advantage of the assumption that the coherent superposition of states for an auxiliary degree of freedom manipulable by Eve does not play a role throughout the measurement process. (This assumption can be justified if in the setup for time-bin measurements there is no quantum interference between any pair of states for the auxiliary degree of freedom manipulable by Eve—which is true in practice as we think.) Therefore, each measurement operator on a single photon is block-diagonal with respect to various states for the auxiliary degrees of freedom, where each block is described by a qubit measurement. As a consequence, we need only to characterize the dependence of the qubit measurement operators on t_1 and t_2, even if t_1 and t_2 are manipulable by Eve. For this, we utilize the relations between the creation operators of the input and output modes of a beam splitter. Specifically, if a beam splitter has a transmission coefficient t with two input modes $1_{\rm in}$ and $2_{\rm in}$ and two output modes $1_{\rm out}$ and $2_{\rm out}$, where a photon in the mode $1_{\rm in}$ is transmitted to the mode $1_{\rm out}$ with probability t and is reflected to the mode $2_{\rm out}$ with probability $(1-t)$, then the creation operators of the output modes are related with those of the input modes by

$$\hat{a}^\dagger_{1_{\rm out}} = \sqrt{t}\hat{a}^\dagger_{1_{\rm in}} + \sqrt{1-t}\hat{a}^\dagger_{2_{\rm in}} \text{ and } \hat{a}^\dagger_{2_{\rm out}} = -\sqrt{1-t}\hat{a}^\dagger_{1_{\rm in}} + \sqrt{t}\hat{a}^\dagger_{2_{\rm in}}. \quad (42)$$

By exploiting such relations for the two beam splitters BS1 and BS2 in the MZI, we obtain the measurement operators,

represented in the single-photon input-mode basis $\{|e\rangle, |l\rangle\}$, as follows:

$$
\begin{aligned}
M_e = M_{e,a} + M_{e,b} &= \begin{bmatrix} t_1 & 0 \\ 0 & 0 \end{bmatrix}, M_l = M_{l,a} + M_{l,b} = \begin{bmatrix} 0 & 0 \\ 0 & r_1 \end{bmatrix}, \\
M_{m,a} &= \begin{bmatrix} r_1 r_2 & \sqrt{r_1 r_2 t_1 t_2} \\ \sqrt{r_1 r_2 t_1 t_2} & t_1 t_2 \end{bmatrix}, M_{m,b} = \begin{bmatrix} r_1 t_2 & -\sqrt{r_1 r_2 t_1 t_2} \\ -\sqrt{r_1 r_2 t_1 t_2} & t_1 r_2 \end{bmatrix},
\end{aligned} \tag{43}
$$

where $r_1 = 1 - t_1$ and $r_2 = 1 - t_2$. Note that the sum of the above four operators is equal to the identity operator, meaning that only click events are observed when the detection efficiency is perfect and the input contains a single photon.

Suppose that $t_i = 1/2 + \tau_i$, $i = 1, 2$, where τ_1 and τ_2 characterize the imperfections of the two beam splitters BS1 and BS2. Then, from Eq. (43) we get the following inequalities

$$
(1/2 - |\tau_1|)\mathbb{1}_2 \leq M_e + M_l, M_{m,a} + M_{m,b} \leq (1/2 + |\tau_1|)\mathbb{1}_2, \tag{44}
$$

where $\mathbb{1}_2$ is the identity matrix of 2×2. Therefore, if we assume that the events e and l are due to the time-bin Z-basis measurement and that the events (m, a) and (m, b) are due to the superposition X-basis measurement on the input time-bin qubit, then the probabilities P_X and P_Z of selecting the X- and Z-bases at a trial satisfy that

$$
(1/2 - |\tau_1|) \leq P_X, P_Z \leq (1/2 + |\tau_1|). \tag{45}
$$

When the event e or (m, a) happens, we specify the outcome as $+1$; when the event l or (m, b) happens, the outcome is -1. With these outcome assignments, the (unnormalized) input-dependent measurement operators in the X- and Z-bases are

$$
\begin{aligned}
M'_X = M_{m,a} - M_{m,b} &= \begin{bmatrix} r_1(r_2 - t_2) & 2\sqrt{r_1 r_2 t_1 t_2} \\ 2\sqrt{r_1 r_2 t_1 t_2} & t_1(t_2 - r_2) \end{bmatrix} = 2\tau_1\tau_2\mathbb{1}_2 + 0.5\sqrt{(1 - 4\tau_1^2)(1 - 4\tau_2^2)}\sigma_1 - \tau_2\sigma_3, \\
M'_Z = M_e - M_l &= \begin{bmatrix} t_1 & 0 \\ 0 & -r_1 \end{bmatrix} = \tau_1\mathbb{1}_2 + 0.5\sigma_3,
\end{aligned} \tag{46}
$$

where $\sigma_1 = \begin{bmatrix} 0 & 1 \\ 1 & 0 \end{bmatrix}$ and $\sigma_3 = \begin{bmatrix} 1 & 0 \\ 0 & -1 \end{bmatrix}$ are two of the Pauli matrices. In view of the above operators and according to Property 3 of Ref. [3], for constructing PEFs and QEFs we can use the classical and quantum models induced by the (unnormalized) input-dependent measurement operators

$$
\tilde{M}'_X = 0.5\sqrt{(1 - 4\tau_1^2)(1 - 4\tau_2^2)}\sigma_1 - \tau_2\sigma_3, \tilde{M}'_Z = 0.5\sigma_3. \tag{47}
$$

The corresponding (normalized) input-dependent measurement operators are

$$
M_X = \sin(\theta)\sigma_1 + \cos(\theta)\sigma_3, M_Z = \sigma_3. \tag{48}
$$

where $\theta = \cos^{-1}\big(-2\tau_2/\sqrt{4\tau_2^2 + (1 - 4\tau_1^2)(1 - 4\tau_2^2)}\big)$. So, the two measurements along the X- and Z-bases can be represented by two directions $\mathbf{n}_X$ and $\mathbf{n}_Z$ on the Bloch sphere, where $\mathbf{n}_X = (\sin(\theta), 0, \cos(\theta))$ and $\mathbf{n}_Z = (0, 0, 1)$. Note that even if the imperfections τ_1 and τ_2 are not exactly known but can only be bounded, we can still bound the probabilities P_X and P_Z as well as the angle θ. Therefore, we allow Eve to manipulate the imperfections in the two beam splitters used via side-channel attacks, as long as the manipulations satisfy the assumed imperfection bounds.

In a word, the imperfection in the first beam splitter BS1 determines the imbalance, $\tau = (P_X - P_Z)/2$, between the probabilities P_X and P_Z of selecting the X- and Z-bases, and the imperfections in both beam splitters BS1 and BS2 affect the misalignment angle, $\delta = \max_\theta |\theta - \pi/2|$.

Case 2. We assume that the two detectors at the output ports a and b have efficiencies η_a and η_b, which are independent of the temporal modes of the incoming photons but can be controlled by Eve via manipulating other degrees of freedom of the photons. For the same reason as in Case 1, we need only to characterize the dependence of the qubit measurement operators on the transmission coefficients t_1 and t_2 and on the efficiencies η_a and η_b, even if these parameters are manipulable by Eve. A practical detector with efficiency η can be described as a beam splitter with transmission coefficient η followed by an ideal detector with perfect efficiency [9]. In view of this fact and by

exploiting the relations in Eq. (42) for the two beam splitters in the MZI as well as for the beam splitters describing the two practical detectors, we get the following measurement operators represented in the single-photon input-mode basis $\{|e\rangle, |l\rangle\}$

$$\begin{aligned} M_e = M_{e,a} + M_{e,b} &= \begin{bmatrix} t_1(\eta_a t_2 + \eta_b r_2) & 0 \\ 0 & 0 \end{bmatrix}, M_l = M_{l,a} + M_{l,b} = \begin{bmatrix} 0 & 0 \\ 0 & r_1(\eta_b t_2 + \eta_a r_2) \end{bmatrix}, \\ M_{m,a} = \eta_a \begin{bmatrix} r_1 r_2 & \sqrt{r_1 r_2 t_1 t_2} \\ \sqrt{r_1 r_2 t_1 t_2} & t_1 t_2 \end{bmatrix}&, M_{m,b} = \eta_b \begin{bmatrix} r_1 t_2 & -\sqrt{r_1 r_2 t_1 t_2} \\ -\sqrt{r_1 r_2 t_1 t_2} & t_1 r_2 \end{bmatrix}, \end{aligned} \tag{49}$$

where the parameters t_1, t_2, r_1 and r_2 are the same as those in the first case. Note that the sum of the above four operators is less than the identity operator in general, since there are no-click events besides the above click events.

Suppose that $t_i = 1/2 + \tau_i$, $i = 1, 2$, and that $\eta_a = \eta + \Delta\eta$ and $\eta_b = \eta - \Delta\eta$, where $\eta = (\eta_a + \eta_b)/2$ is the averaged detection efficiency of the two detectors employed and $\Delta\eta$ characterizes the mismatch between η_a and η_b. Then, from Eq. (49) we get the following expressions

$$\begin{aligned} M_e - M_l &= \eta(\lambda_0 \mathbb{1}_2 + \lambda_3\sigma_3), \\ M_{m,a} - M_{m,b} &= \eta(\chi_0 \mathbb{1}_2 + \chi_1\sigma_1 + \chi_3\sigma_3), \end{aligned} \tag{50}$$

and

$$\begin{aligned} M_e + M_l &= \eta(\lambda'_0 \mathbb{1}_2 + \lambda'_3\sigma_3), \\ M_{m,a} + M_{m,b} &= \eta(\chi'_0 \mathbb{1}_2 + \chi'_1\sigma_1 + \chi'_3\sigma_3), \end{aligned} \tag{51}$$

with coefficients given by

$$\begin{aligned} &\lambda_0 = \lambda'_3 = \tau_1 + \tau_2\Delta\eta/\eta, \lambda_3 = \lambda'_0 = \chi'_0 = 0.5 + 2\tau_1\tau_2\Delta\eta/\eta \geq 0, \\ &\chi_0 = 0.5\Delta\eta/\eta + 2\tau_1\tau_2, \chi_1 = 0.5\sqrt{(1-4\tau_1^2)(1-4\tau_2^2)}, \chi_3 = -\tau_1\Delta\eta/\eta - \tau_2, \\ &\chi'_1 = 0.5\Delta\eta/\eta\sqrt{(1-4\tau_1^2)(1-4\tau_2^2)} = \Delta\eta/\eta\chi_1, \text{ and } \chi'_3 = -\tau_1 - \tau_2\Delta\eta/\eta = -\lambda'_3. \end{aligned} \tag{52}$$

Suppose that the events e and l are due to the time-bin Z-basis measurement and that the events (m, a) and (m, b) are due to the superposition X-basis measurement. According to Eq. (51), the probabilities $\tilde{P}_X$ and $\tilde{P}_Z$ of selecting the X- and Z-bases at a trial satisfy

$$\begin{aligned} \eta(\chi'_0 - \sqrt{|\chi'_1|^2 + |\chi'_3|^2}) = \Lambda_{\min}\big(M_{m,a} + M_{m,b}\big) &\leq \tilde{P}_X \leq \Lambda_{\max}\big(M_{m,a} + M_{m,b}\big) = \eta(\chi'_0 + \sqrt{|\chi'_1|^2 + |\chi'_3|^2}), \\ \eta(\lambda'_0 - |\lambda'_3|) = \Lambda_{\min}\big(M_e + M_l\big) &\leq \tilde{P}_Z \leq \Lambda_{\max}\big(M_e + M_l\big) = \eta(\lambda'_0 + |\lambda'_3|), \\ \eta(\lambda'_0 + \chi'_0 - |\chi'_1|) = \Lambda_{\min}\big(M_e + M_l + M_{m,a} + M_{m,b}\big) &\leq \tilde{P}_X + \tilde{P}_Z \leq \Lambda_{\max}\big(M_e + M_l + M_{m,a} + M_{m,b}\big) = \eta(\lambda'_0 + \chi'_0 + |\chi'_1|), \end{aligned} \tag{53}$$

where for a matrix M, $\Lambda_{\max}(M)$ and $\Lambda_{\min}(M)$ denote its maximum and minimum eigenvalues. In general, the probabilities $\tilde{P}_X$ and $\tilde{P}_Z$ are unnormalized as there are no-click events. Define the normalized probabilities as $P_X = \tilde{P}_X/(\tilde{P}_X + \tilde{P}_Z)$ and $P_Z = \tilde{P}_Z/(\tilde{P}_X + \tilde{P}_Z)$. Then P_X and P_Z satisfy

$$\begin{aligned} \frac{\chi'_0 - \sqrt{|\chi'_1|^2 + |\chi'_3|^2}}{\lambda'_0 + \chi'_0 + |\chi'_1|} &\leq P_X \leq \frac{\chi'_0 + \sqrt{|\chi'_1|^2 + |\chi'_3|^2}}{\lambda'_0 + \chi'_0 - |\chi'_1|}, \\ \frac{\lambda'_0 - |\lambda'_3|}{\lambda'_0 + \chi'_0 + |\chi'_1|} &\leq P_Z \leq \frac{\lambda'_0 + |\lambda'_3|}{\lambda'_0 + \chi'_0 - |\chi'_1|}. \end{aligned} \tag{54}$$

Therefore, the bounds on the imbalance $\tau = (P_X - P_Z)/2$ are

$$\tau_{\text{ub}} = \frac{1}{2}\Big(\frac{\chi'_0 + \sqrt{|\chi'_1|^2 + |\chi'_3|^2}}{\lambda'_0 + \chi'_0 - |\chi'_1|} - \frac{\lambda'_0 - |\lambda'_3|}{\lambda'_0 + \chi'_0 + |\chi'_1|}\Big),$$

$$\tau_{\text{lb}} = \frac{1}{2}\Big(\frac{\chi'_0 - \sqrt{|\chi'_1|^2+|\chi'_3|^2}}{\lambda'_0+\chi'_0+|\chi'_1|} - \frac{\lambda'_0+|\lambda'_3|}{\lambda'_0+\chi'_0-|\chi'_1|}\Big). \tag{55}$$

By the same argument as in the first case and in view of Eq. (50), for constructing PEFs and QEFs we can use the classical and quantum models induced by the (normalized) input-dependent measurement operators

$$M_X = \sin(\theta)\sigma_1 + \cos(\theta)\sigma_3, M_Z = \sigma_3. \tag{56}$$

where $\theta = \cos^{-1}(\chi_3/\sqrt{\chi_1^2+\chi_3^3})$. So, like in the first case, the two measurements along the X- and Z-bases can be represented by two directions $\mathbf{n}_X$ and $\mathbf{n}_Z$ on the Bloch sphere, where $\mathbf{n}_X = (\sin(\theta), 0, \cos(\theta))$ and $\mathbf{n}_Z = (0,0,1)$. Note that even if the imperfections τ_1, τ_2 and $\Delta\eta/\eta$ are not exactly known but can only be bounded, we can still bound the probabilities P_X and P_Z as well as the angle θ. Therefore, in this case we allow Eve to manipulate, via side-channel attacks, the efficiency mismatch in addition to the imperfections in the two beam splitters used, as long as the manipulations satisfy the assumed imperfection bounds. Also, in contrast to the first case, here both the imbalance $\tau = (P_X - P_Z)/2$ and the misalignment angle $\delta = \max_\theta |\theta - \pi/2|$ depend on all the considered imperfections τ_1, τ_2 and $\Delta\eta/\eta$.

To calibrate the imperfections in our measurement apparatus, we send a weak pulse in the early time-bin mode, the same as the pulse used for randomness generation, into the MZI. The pulse can arrive at the output of the MZI in the early (e) or middle (m) time-bin mode, and then be detected by a threshold detector at either the output port a or b. We perform the calibration in the absence of Eve's manipulation, and we assume that the parameters to be calibrated are stable over time. Specifically, we assume that the two detectors at the output ports a and b have efficiencies η_a and η_b, and that the two beam splitters BS1 and BS2 in the MZI have transmission coefficients t_1 and t_2, respectively. In order to handle the cases where the parameters to be calibrated fluctuate over time or can be manipulated by Eve via side-channel attacks, we later set conservative bounds on the calibrated imperfections for performing security analysis. The probabilities of detections at each possible output mode (a combination of the temporal and spatial modes) depend on the parameters t_1, t_2, η_a and η_b, as shown in Supplementary Table I(1). Therefore, we can estimate these parameters based on experimental results. In particular, we used 360 s of experimental data for calibration. Denote the number of counts by the detector a for the early time-bin mode e of the output over 360 s of experiment time by $n(a,e)$; the numbers of counts at the other output modes are denoted in a similar way. These results are presented in Supplementary Table I(2). In view of the fact that the number of counts at an output mode is proportional to the corresponding detection probability, we obtain the following relations

$$\sqrt{\frac{n(a,e)}{n(b,m)}\frac{n(b,e)}{n(a,m)}} = \frac{t_1}{1-t_1} \text{ and } \sqrt{\frac{n(a,e)}{n(b,e)}\frac{n(b,m)}{n(a,m)}} = \frac{t_2}{1-t_2}. \tag{57}$$

Plugging the experimental results in Supplementary Table I(2) into the above relations, we get $t_1 = 0.462$ and $t_2 = 0.531$. So, the splitting ratios between the reflection and transmission coefficients for the two beam splitters BS1 and BS2 in the MZI are 53.8:46.2 and 46.9:53.1, respectively. In addition, we can estimate the mismatch between the efficiencies η_a and η_b of the two detectors employed. Specifically, in view of Supplementary Table I(1) we have

$$\frac{n(a,e)+n(a,m)}{n(b,e)+n(b,m)} = \frac{\eta_a}{\eta_b}\frac{t_1t_2+(1-t_1)(1-t_2)}{t_1(1-t_2)+(1-t_1)t_2}. \tag{58}$$

Combining Eqs. (57) and (58) and then plugging the experimental results in Supplementary Table I(2), we get that $\eta_a/\eta_b = 1.024$ and so $\Delta\eta/\eta = 0.0119$. This estimated efficiency-mismatch ratio is consistent with the detection efficiencies $\eta_a = (60 \pm 2)\%$ and $\eta_b = (59 \pm 2)\%$ measured before the experiment. This estimation suggests the potential presence of efficiency mismatch in our measurement setup.

Now we can translate the above estimated imperfections in our measurement apparatus into the imperfection bounds required by our security-analysis method. If we consider the first case discussed above, where the efficiency mismatch is assumed to be absent, then according to Eqs. (45) and (48) we estimate that $|\tau| \le 0.038$ and $\delta \le 3.565°$. If we consider the second case where the effect of efficiency mismatch is included, then according to Eqs. (55) and (56) we estimate that $|\tau| \le 0.041$ and $\delta \le 3.513°$. In view of these estimated bounds, we conservatively assume that $|\tau| \le 0.06$ and $\delta \le 6°$ in our security analysis.

We remark that there are several previous works on how to relax assumptions on measurement devices. In particular, the measurement-device-independent (MDI) QRNG schemes proposed in Refs. [10, 11] have the potential to be resistant to all adversarial imperfections in the measurement apparatus including efficiency mismatch. However, a MDI scheme requires that the states prepared are fully characterized, which is difficult to promise in practice. There is also a dimension-witness-based QRNG scheme as developed in Ref. [12]. Such scheme does not require characterizing

Supplementary Table I: Results for calibrating the imperfections in our measurement apparatus. The left table (1) shows the unnormalized probabilities of detections at each possible output mode, while the right table (2) shows the results recorded over 360 s in the experiment for estimating the transmission coefficients t_1 and t_2 of the two beam splitters in the MZI as well as the mismatch between the efficiencies η_a and η_b of the two detectors employed.

(1) Unnormalized detection probabilities		
	early bin e	middle bin m
Detector a	$t_1 t_2 \eta_a$	$(1-t_1)(1-t_2)\eta_a$
Detector b	$t_1(1-t_2)\eta_b$	$(1-t_1)t_2\eta_b$

(2) Counts used for calibrating imperfections		
	early bin e	middle bin m
Detector a	41284545	42500666
Detector b	35574756	47022242

the states prepared or the measurement performed, but it makes an assumption on the dimension of the quantum system used, particularly the assumption that the system is a qubit of dimension two. This assumption is hard to be verified in an experiment, and it is not satisfied if a weak optical pulse is used as in our QRNG. Moreover, recently a QRNG scheme based on energy constraints was proposed [13] and analyzed [14, 15]. In a energy-constraint-based scheme, the average photon number in each possible prepared state is bounded from above, and except these constraints, no other characterization of the states prepared or the measurement performed is needed. The energy constraints are similar to the lower bound $q_{1,\mathrm{lb}}$ on the single-photon probability required by our scheme. From the point view of practical implementations, the assumption behind the energy-constraint-based scheme is more natural than that behind the dimension-witness-based scheme. Compared with the above MDI or dimension-witness-based schemes as well as the source-independent schemes [16, 17], our scheme achieves a reasonable compromise between the adversarial imperfections in the state source and those in the measurement apparatus. Compared with the energy-constraint-based scheme, our scheme takes advantage of the bounds on the adversarial imperfections in the measurement apparatus and so can achieve better performance for randomness generation. Also, the security analyses in previous works are not as general as ours. Especially, the security analysis for the dimension-witness-based QRNG in Ref. [12] or for the energy-constraint-based QRNG in Ref. [14] works only in the presence of classical side information.

B. Imperfections in photon source

At each trial the photon source emits zero photon or multiple photons with non-zero probabilities. Like for quantum key distribution [18], zero-photon events do not affect the security analysis of randomness generation but only the randomness-generation rate and latency achieved in practice. Strictly speaking, zero-photon events affect the bound $q_{1,\mathrm{lb}}$ used in our model constructions (see below) but not the validity of our security-analysis method. So, we can focus on the treatment of multiphoton events. There are two effects caused by multiphoton events. First, in the constructions of both the classical model $\mathcal{C}$ and the quantum model $\mathcal{Q}$ for a trial, we need to consider the consequence of multiphoton events. For this, we introduced a lower bound $q_{1,\mathrm{lb}}$ on the single-photon probability. Suppose that the probabilities of emitting a single photon and multiple photons from the photon source are p_1 and $p_{\geq 2}$ respectively. Then, as zero-photon events are not relevant for security analysis, the single-photon probability lower bound $q_{1,\mathrm{lb}}$ for model constructions can be set to a value as large as $p_1/(p_1+p_{\geq 2})$. In our experiment, the average photon number per trial (that is, per pulse emitted from the source) is 0.0035. So, we estimated that $p_1 = 3.48\times 10^{-3}$ and $p_{\geq 2} = 1.22\times 10^{-5}$ according to the thermal distribution that describes the photon-number statistics of the optical pulse generated in our experiment. Therefore, we can set $q_{1,\mathrm{lb}}$ to be a value as large as 0.997. In our security analysis we conservatively set $q_{1,\mathrm{lb}} = 0.98$. Note that in practice not all photons emitted from the source are detected because of the detection inefficiency and the insertion loss of the MZI. In this case, we need to consider the effect of photon loss on the choice of $q_{1,\mathrm{lb}}$. The details will be presented in the next paragraph, and the result shows that our choice $q_{1,\mathrm{lb}} = 0.98$ is still valid even with an arbitrary photon loss. Second, multiphoton events can cause simultaneous photon detections at more than one of the six output modes as labelled in Supplementary Figure 2 (2). As each measurement along the X- or Z-basis is assumed to have two outcomes ± 1 for constructing the models $\mathcal{C}$ and $\mathcal{Q}$, before the experiment we need to fix the assignments of the simultaneous photon detections to the outcomes $+1$ or -1 at some basis. We emphasize that there are freedoms in such assignments as long as they are fixed before the experiment. Specifically, in our security analysis these assignments are as follows: 1) The simultaneous photon detections (that is, double clicks) in the Z-basis are mapped to the outcome $+1$ or -1 in the same basis. Specifically, the double clicks at the output modes (e,a) and (e,b) are mapped to $+1$, the double clicks at the output modes (l,a) and (l,b) are mapped to -1, and the double clicks at both the early and late time-bin modes (for example, the clicks at both output modes (e,a) and (l,b)) are mapped to -1. 2) The simultaneous photon detections in the X-basis, that is, the double clicks at the output modes (m,a) and (m,b), are mapped to the outcome -1 in the

same basis. 3) The simultaneous photon detections at the X- and Z-bases (that is, cross clicks), which include the simultaneous detections at the early and middle time-bin modes, the simultaneous detections at the middle and late time-bin modes, and the simultaneous detections at all the three time-bin modes, are all mapped to the outcome -1 in the X-basis.

Several remarks are as follows. First, we allow Eve to manipulate the distribution of photon numbers emitted from the source, as long as Eve's manipulation satisfies the lower bound $q_{1,\mathrm{lb}}$ used in our security analysis. Second, because of the detection inefficiency or the insertion loss of the MZI in practice, the single-photon or multiphoton components of the optical pulse are not always detected. For security analysis, we make the fair-sampling assumption that the detected sub-ensemble is a fair representation of the whole ensemble. Therefore, the same as zero-photon events, the no-click events due to photon loss do not affect our security analysis but only the randomness-generation rate and latency achieved in practice. Strictly speaking, the no-click events due to photon loss does not affect the validity of our security-analysis method, but they affect the bound $q_{1,\mathrm{lb}}$ used in our model constructions which is detailed as follows. Suppose that the probability of emitting j photons from the photon source is p_j, $j = 0, 1, 2, \ldots$, and that the total photon loss in the MZI and in the detection process is ξ, where $\xi \in [0, 1)$. Note that ξ is the probability that a single photon is lost in the process of measurement and detection. Then, the detector-click probability conditional on the event that j photons are emitted from the source is $p(\mathrm{click}|j) = 1 - \xi^j$. Therefore, the probability of the joint event that a detector clicks and j photons are emitted is $p(\mathrm{click}, j) = p_j(1 - \xi^j)$. So, the lower bound $q_{1,\mathrm{lb}}$ for model constructions should satisfy

$$\begin{aligned} q_{1,\mathrm{lb}} &\leq \frac{p(\mathrm{click}, j=1)}{\sum_{j\geq 1} p(\mathrm{click}, j)} = \frac{p_1 p(\mathrm{click}|j=1)}{\sum_{j\geq 1} p_j p(\mathrm{click}|j)} \\ &= \frac{p_1(1-\xi)}{p_1(1-\xi) + \sum_{j\geq 2} p_j p(\mathrm{click}|j)} \\ &= \frac{p_1}{p_1 + \sum_{j\geq 2} p_j f_j(\xi)}, \end{aligned} \tag{59}$$

where $f_j(\xi) = p(\mathrm{click}|j)/(1-\xi) = (1-\xi^j)/(1-\xi)$. When $j \geq 2$, the factor $f_j(\xi)$ is a monotonically increasing function of $\xi \in [0, 1)$ (by checking the positivity of the first derivative of $f_j(\xi)$ when $\xi \in [0, 1)$). Accordingly, we have

$$q_{1,\mathrm{lb}} \leq \frac{p_1}{p_1 + \sum_{j\geq 2} p_j \lim_{\xi\to 1^-} f_j(\xi)} = \frac{p_1}{p_1 + \sum_{j\geq 2} p_j j} = \frac{p_1}{\mu}, \tag{60}$$

where $\mu = \sum_{j\geq 1} j p_j$ is the average photon number in a pulse emitted from the source. In our experiment, $\mu = 0.0035$ and $p_1 = 3.48 \times 10^{-3}$ (see the above paragraph). So, we can set $q_{1,\mathrm{lb}}$ to be a value as large as 0.993 regardless of the specific photon loss, while in our security analysis we used the more conservative choice $q_{1,\mathrm{lb}} = 0.98$. Third, our treatment of multiphoton events is different from the treatments in Refs. [16, 19–21]. These references rely on a particular post-processing map—a squashing map that assigns a double-click event uniformly randomly to be one of the two single-click events at the same measurement basis and assigns all cross-click events to no detection. After applying the squashing map, one can prove that for some particular measurements (for example, when the X- and Z-bases are mutually unbiased and when there is no detection-efficiency mismatch), the distribution of measurement outcomes according to a multiphoton state can be reproduced by the same measurements on some single-photon state. Therefore, with a squashing map the measurement outcomes according to a multiphoton state can be treated in the same way as those according to a single-photon state, and so the security analysis taking into account multiphoton events can be reduced to the security analysis in the presence of only single-photon events. In contrast, we treat the measurement outcomes according to a multiphoton state in a different way: We explicitly separate the contribution of multiphoton events from that of single-photon events when constructing the models $\mathcal{C}$ and $\mathcal{Q}$, and for the multiphoton part we conservatively assume that the measurements are un-characterized as long as each measurement has only two outcomes (after applying our strategy for assigning simultaneous photon detections as specified in the previous paragraph). So, our treatment works for arbitrary measurements, unlike the squashing map [16, 19–21] which exists only for ideal measurements without any misalignment or efficiency mismatch. Another advantage is that the outcome assignments for simultaneous photon detections used by us do not consume additional random numbers as compared with the squashing map used in Refs. [16, 19–21].

Supplementary Note 4. Analysis details for randomness generation

The goal of an instance of our QRNG is to generate $k_{\mathrm{q}} = 8192$ (or $k_{\mathrm{c}} = 2\times 8192$) random bits with soundness error $\epsilon = 2^{-64}$ in the presence of quantum (or classical) side information. For this, we perform analysis according to the following steps, which are also summarized in Box. 1.

Box 1: Overview of data analysis in the presence of quantum (or classical) side information

1. Parameter determination: Fix the parameters p and ϵ_s in Theorem 2 (or Theorem 1) of the main text.
2. Calibration
   (a) Determine the QEF $F_{\mathrm{q}}(I,O)$ and its power β_{q} (or the PEF $F_{\mathrm{c}}(I,O)$ and its power β_{c}) to be used for each executed trial.
   (b) Fix n_{q} (or n_{c}) — the maximum number of trials that can be used for randomness accumulation.
3. Randomness Accumulation: Run the experiment to acquire up to n_{q} (or n_{c}) trials. After each trial m,
   (a) Update the running value $T_{\mathrm{q},m} = \prod_{k=1}^{m} F_{\mathrm{q}}(i_k,o_k)$ (or $T_{\mathrm{c},m} = \prod_{k=1}^{m} F_{\mathrm{c}}(i_k,o_k)$).
   (b) If $T_{\mathrm{q},m} \geq 1/\big(p^{\beta_{\mathrm{q}}}(\epsilon_s^2/2)\big)$ (or $T_{\mathrm{c},m} \geq 1/\big(p^{\beta_{\mathrm{c}}}\epsilon_s\big)$), stop the experiment, set the number of trials actually executed as $n_{\mathrm{q,act}} = m$ (or $n_{\mathrm{c,act}} = m$), and claim the success of the instance.

   If $\max_{m\leq n_{\mathrm{q}}} T_{\mathrm{q},m} < 1/\big(p^{\beta_{\mathrm{q}}}(\epsilon_s^2/2)\big)$ (or $\max_{m\leq n_{\mathrm{c}}} T_{\mathrm{c},m} < 1/\big(p^{\beta_{\mathrm{c}}}\epsilon_s\big)$), the instance fails.
4. Randomness Extraction: Conditional on success, run the TMPS extractor to generate k_{q} (or k_{c}) random bits with error ϵ.

The first step is to set the parameters required for our analysis. To achieve the goal, we set the smoothness error to be $\epsilon_s = 0.8\epsilon \approx 4.34\times 10^{-20}$ and the extractor error to be $\epsilon_x = 0.2\epsilon \approx 1.08\times 10^{-20}$. We emphasize that the positive errors ϵ_s and ϵ_x need to satisfy that $\epsilon_s+\epsilon_x \leq \epsilon$, but their choices are not unique. The splitting ratio 0.8:0.2 between ϵ_s and ϵ_x used by us was not optimized; instead it was chosen heuristically for the same reason as in Ref. [5]. Moreover, to achieve our goal with the TMPS extractor [22, 23], the amount of quantum (or classical) ϵ_s-smooth conditional min-entropy to be certified (without taking into account the probability of success) is 8516 (or 16712) bits. According to Theorem 2 (or Theorem 1) of the main text, the amount of quantum (or classical) ϵ_s-smooth conditional min-entropy certified in a successful QRNG instance (without taking into account the probability of success) is bounded from below by $-\log_2(p)$. Consequently, for our goal it suffices to set the parameter p in Theorem 2 (or Theorem 1) of the main text to be 2^{-8516} (or 2^{-16712}).

The second step is calibration based on the data obtained prior to the data used for randomness generation. The data sets for calibration and randomness generation are called the calibration data and the analysis data, respectively. As a result of this step, we determined a well-performing QEF $F_{\mathrm{q}}(I,O)$ and its power β_{q} (or a well-performing PEF $F_{\mathrm{c}}(I,O)$ and its power β_{c}) to be used for each executed trial for randomness generation, and fixed the maximum number of trials n_{q} (or n_{c}) that can be used for each QRNG instance, see Supplementary Note 4 A for details.

In the calibration step, we also estimated that a QRNG instance with a high probability of success requires about 0.1 s runtime (see Supplementary Note 4 A for details). Considering that we have 420 s of data for randomness generation, we thus decided ahead of time to run 4200 instances of our QRNG. We also decided to use the same QEF (or PEF) as well as the corresponding maximum number of trials n_{q} (or n_{c}) for all instances. That is, when we ran 4200 instances of our QRNG, we performed the calibration step only once before processing all 420 s of data for randomness generation. In this way, the time cost for calibration would not contribute to the latency in randomness generation.

The third step consists of acquiring up to n_{q} (or n_{c}) trials. After each trial m, we update the running value of the QEF product $T_{\mathrm{q},m} = \prod_{k=1}^{m} F_{\mathrm{q}}(i_k,o_k)$ (or the running value of the PEF product $T_{\mathrm{c},m} = \prod_{k=1}^{m} F_{\mathrm{c}}(i_k,o_k)$). Both QEFs and PEFs have the advantage that one can stop the experiment early as soon as the running value $T_{\mathrm{q},m}$ (or $T_{\mathrm{c},m}$) surpasses the corresponding success threshold $1/\big(p^{\beta_{\mathrm{q}}}(\epsilon_s^2/2)\big)$ in Theorem 2 (or $1/\big(p^{\beta_{\mathrm{c}}}\epsilon_s\big)$ in Theorem 1) of the main text. This early-stopping feature is due to the fact that the constant function $F(i,o)=1$ for all inputs i and outputs o of a trial is both a PEF with any positive power for all classical models and a QEF with any positive power for all quantum models, see Refs. [1–4] for more detailed explanations. If for each possible m, $T_{\mathrm{q},m}$ (or $T_{\mathrm{c},m}$) fails to surpass the corresponding success threshold, the instance of our QRNG fails. When we ran the analysis, we found that each

instance succeeded before the data block used for the instance was completely processed, see Supplementary Note 4 B for details. Let $n_{\mathrm{q,act}}$ (or $n_{\mathrm{c,act}}$) be the actual number of trials executed.

The fourth and last step consists of running the TMPS extractor with the trial outputs (when the instance succeeds). The extractor input is exactly n_{q} bits (or n_{c} bits) long and consists of the trial outputs padded with zeros to n_{q} bits if $n_{\mathrm{q,act}} < n_{\mathrm{q}}$ (or padded with zeros to n_{c} bits if $n_{\mathrm{c,act}} < n_{\mathrm{c}}$). The number of seed bits required for running the extractor is determined by the length of the extractor input, the number of random bits to be extracted and the extractor error as instructed in Refs. [2, 4, 5, 24]. In each QRNG instance, to extract 8192 (or 2×8192) random bits with error 2^{-64} in the presence of quantum (or classical) side information the number of seed bits required is 1031048 (or 1288810). In our numerical implementation of the TMPS extractor, the extraction of 8192 (or 2×8192) random bits took about 0.02 s (or 0.04 s) on a cluster with 30 threads. We remark that as the TMPS extractor is a strong extractor, the extractor seed could be reused for the future instance of our QRNG as long as the extractor seed is independent of the other random variables or quantum systems involved in the future instance; however, the seed bits to be reused would have a statistical distance from uniform that needs to be added in order to adjust the soundness error claimed in the future instance.

A. Calibration details

For the calibration purpose, we took 360 s of experimental data (see Supplementary Table II(1)). This calibration data is the same as that used for calibrating the imperfections in our measurement apparatus and shown in Supplementary Table I(2); however, the same data is represented in different forms in Supplementary Table I(2) and in Supplementary Table II(1). With the calibration data shown in Supplementary Table II(1), we can get an estimate of the distribution of inputs and outputs at an experimental trial. The estimated distribution is denoted by $\mathbb{P}_{\mathrm{est}}(I, O)$, which is given by the relative frequency distribution of the processed results presented in Supplementary Table II(2). Also, based on the calibration data, we observed that every 0.1 s runtime the experiment generates about 47000 trials with clicks. To determine a well-performing QEF $F_{\mathrm{q}}(I, O)$ and its power β_{q} (or a well-performing PEF $F_{\mathrm{c}}(I, O)$ and its power β_{c}), we solved the QEF optimization of Eq. (40) (or the PEF optimization of Eq. (10)) with the parameters $n = 47000$, $\epsilon_s = 0.8\epsilon \approx 4.34 \times 10^{-20}$, and the replacement of the distribution $\mathbb{P}_{\mathrm{exp}}(I, O)$ by the above estimated distribution $\mathbb{P}_{\mathrm{est}}(I, O)$. The solutions of these two optimization problems are presented in Supplementary Table III. Note that to obtain these solutions, both the quantum and classical models used for each experimental trial are constructed in the presence of the adversarial imperfections specified in Supplementary Note 3.

Supplementary Table II: Counts of results used for calibration. The left table (1) shows the counts of raw results recorded over 360 s in the experiment, and the right table (2) shows the counts of the corresponding processed results which are obtained according to the strategy specified in the second last paragraph of Supplementary Note 3 for assigning double clicks and cross clicks to the outcomes +1 or −1.

(1) Counts of raw results				
	+1	−1	double clicks	cross clicks
Z-basis	76828705	3194445	711	0
X-basis	46996639	42477589	14239	0
both bases	0	0	0	20200

(2) Counts of processed results		
	+1	−1
Z-basis	76828705	3215356
X-basis	46996639	42491828

Supplementary Table III: The QEF and PEF used for our QRNG

The QEF $F_{\mathrm{q}}(I, O)$ with power $\beta_{\mathrm{q}} = 0.095$		
I \ O	+1	−1
Z-basis	1.014852783106925	0.744083927729296
X-basis	1.041632513459442	1.041632513459442

The PEF $F_{\mathrm{c}}(I, O)$ with power $\beta_{\mathrm{c}} = 0.099$		
I \ O	+1	−1
Z-basis	1.000970783946250	0.932084097129438
X-basis	1.068539312040006	1.068539312040006

With the QEF and PEF obtained above, we can also estimate the probability that every 0.1 s runtime our QRNG successfully generates 8192 (or 2×8192) random bits with soundness error 2^{-64} in the presence of quantum (or

classical) side information. For the estimates, we assume that the quantum device to be used for our QRNG is honest with the distribution $\mathbb{P}_{\text{est}}(I,O)$ for each trial. Then, by Hoeffding's inequality [25], we found that the success probability is at least $1-2^{-380}$ (or $1-2^{-478}$) using the QEF (or PEF) in Supplementary Table III. Therefore, the goal specified for each QRNG instance can be satisfied within 0.1 s runtime with an extremely high success probability. For randomness generation, we thus decided ahead of time to use the data block obtained in 0.1 s runtime for each instance, and set the maximum number of trials allowed for the instance to an upper bound on the number of trials with clicks collected over 0.1 s runtime. Particularly, we set $n_{\text{q}} = n_{\text{c}} = 50000$.

B. Analysis results

In view of the early-stopping property of both QEFs and PEFs, when running the data analysis we found that each QRNG instance succeeded before the data block obtained in 0.1 s runtime was completely processed. Let the number of trials with clicks collected over 0.1 s runtime be n, which depends on the instance slightly but is always smaller than $n_{\text{q}} = n_{\text{c}} = 50000$. After the success event happened, we continued to process the remainder of the data observed in each 0.1 s runtime. Finally, we obtained the value for $T_{\text{q},n}$ (or $T_{\text{c},n}$) according to the description in Box. 1. By Theorem 2 (or Theorem 1) of the main text, the amount of quantum (or classical) ϵ_s-smooth conditional min-entropy certified in a successful instance (without taking into account the probability of success) is at least $-\log_2(p)$, where $-\log_2(p) \leq \log_2(T_{\text{q},n})/\beta_{\text{q}} + \log_2(\epsilon_s^2/2)/\beta_{\text{q}}$ (or $-\log_2(p) \leq \log_2(T_{\text{c},n})/\beta_{\text{c}} + \log_2(\epsilon_s)/\beta_{\text{c}}$). Therefore, we set the maximum amount of certifiable quantum (or classical) ϵ_s-smooth conditional min-entropy to be $\log_2(T_{\text{q},n})/\beta_{\text{q}} + \log_2(\epsilon_s^2/2)/\beta_{\text{q}}$ (or $\log_2(T_{\text{c},n})/\beta_{\text{c}} + \log_2(\epsilon_s)/\beta_{\text{c}}$). Then we determined the (maximum) number of random bits extractable using the TMPS extractor with error $\epsilon_x = 0.2 \times 2^{-64}$ in the presence of quantum (or classical) side information. The results for all 4200 instances are shown in Figure 4 of the main text.

In the same way as above, we can vary the soundness error ϵ and study the expected number of random bits certifiable from the data observed every 0.1 s runtime. For this, we set the smoothness error to be $\epsilon_s = 0.8\epsilon$ and the extractor error to be $\epsilon_x = 0.2\epsilon$. For each value of ϵ studied, we solved the QEF optimization of Eq. (40) and PEF optimization of Eq. (10) with the parameters $n = 47000$, $\epsilon_s = 0.8\epsilon$, and the replacement of the distribution $\mathbb{P}_{\text{exp}}(I,O)$ by the distribution $\mathbb{P}_{\text{est}}(I,O)$ estimated during calibration. Their solutions are denoted by $F_{\text{q}}(I,O)$ with power β_{q} and $F_{\text{c}}(I,O)$ with power β_{c}, respectively. With these solutions, we set the expected values $\hat{T}_{\text{q},n}$ and $\hat{T}_{\text{c},n}$ according to

$$\log_2(\hat{T}_{\text{q},n}) = \sum_{i,o} n\mathbb{P}_{\text{est}}(I=i,O=o)\log_2(F_{\text{q}}(i,o)),$$
$$\log_2(\hat{T}_{\text{c},n}) = \sum_{i,o} n\mathbb{P}_{\text{est}}(I=i,O=o)\log_2(F_{\text{c}}(i,o)).$$

Then we set the expected value of the maximum amount of certifiable quantum (or classical) ϵ_s-smooth conditional min-entropy to be $\log_2(\hat{T}_{\text{q},n})/\beta_{\text{q}} + \log_2(\epsilon_s^2/2)/\beta_{\text{q}}$ (or $\log_2(\hat{T}_{\text{c},n})/\beta_{\text{c}} + \log_2(\epsilon_s)/\beta_{\text{c}}$). Finally, we obtained the expected number of random bits extractable using the TMPS extractor with error $\epsilon_x = 0.2\epsilon$ in the presence of quantum (or classical) side information. The results are shown in Figure 3 of the main text, where the soundness error ϵ varies from 10^{-5} to 10^{-30}.

[1] Yanbao Zhang, Emanuel Knill, and Peter Bierhorst. Certifying quantum randomness by probability estimation. *Phys. Rev. A*, 98:040304(R), Oct 2018.

[2] E. Knill, Y. Zhang, and P. Bierhorst. Generation of quantum randomness by probability estimation with classical side information. *Phys. Rev. Research*, 2:033465, 2020.

[3] Yanbao Zhang, Honghao Fu, and Emanuel Knill. Efficient randomness certification by quantum probability estimation. *Phys. Rev. Research*, 2:013016, 2020.

[4] Emanuel Knill, Yanbao Zhang, and Honghao Fu. Quantum probability estimation for randomness with quantum side information. arXiv:1806.04553, 2018.

[5] Yanbao Zhang, Lynden K. Shalm, Joshua C. Bienfang, Martin J. Stevens, Michael D. Mazurek, Sae Woo Nam, Carlos Abellán, Waldimar Amaya, Morgan W. Mitchell, Honghao Fu, Carl A. Miller, Alan Mink, and Emanuel Knill. Experimental low-latency device-independent quantum randomness. *Phys. Rev. Lett.*, 124:010505, 2020.

[6] R. L. Frank and E. H. Lieb. Monotonicity of a relative Rényi entropy. *J. Math. Phys.*, 54:122201, 2013.

[7] L. Masanes. Asymptotic violation of Bell inequalities and distillability. *Phys. Rev. Lett.*, 97:050503/1–4, 2006.

[8] S. Pironio, A. Acin, N. Brunner, N. Gisin, S. Massar, and V. Scarani. Device-independent quantum key distribution secure against collective attacks. *New Journal of Physics*, 11:045021, 2009.

[9] Mark Fox. *Quantum Optics: An Introduction.* OUP Oxford, 2006.

[10] Anubhav Chaturvedi and Manik Banik. Measurement-device-independent randomness from local entangled states. *Europhys. Lett.*, 112:30003, 2015.

[11] Zhu Cao, Hongyi Zhou, and Xiongfeng Ma. Loss-tolerant measurement-device-independent quantum random number generation. *New J. Phys.*, 17:125011, 2015.

[12] Tommaso Lunghi, Jonatan Bohr Brask, Charles Ci Wen Lim, Quentin Lavigne, Joseph Bowles, Anthony Martin, Hugo Zbinden, and Nicolas Brunner. Self-testing quantum random number generator. *Phys. Rev. Lett.*, 114:150501, Apr 2015.

[13] Thomas Van Himbeeck, Erik Woodhead, Nicolas J. Cerf, Raul Garcia-Patron, and Stefano Pironio. Semi-device-independent framework based on natural physical assumptions. *Quantum*, 1:33.

[14] Thomas Van Himbeeck and Stefano Pironio. Correlations and randomness generation based on energy constraints. arXiv:1905.09117, 2019.

[15] Davide Rusca, Thomas van Himbeeck, Anthony Martin, Jonatan Bohr Brask, Weixu Shi, Stefano Pironio, Nicolas Brunner, and Hugo Zbinden. Practical self-testing quantum random number generator based on an energy bound. *Phys. Rev. A*, 100:062338, 2019.

[16] Zhu Cao, Hongyi Zhou, Xiao Yuan, and Xiongfeng Ma. Source-independent quantum random number generation. *Phys. Rev. X*, 6:011020, Feb 2016.

[17] Davide G. Marangon, Giuseppe Vallone, and Paolo Villoresi. Source-device-independent ultra-fast quantum random number generation. *Phys. Rev. Lett.*, 118:060503, 2017.

[18] Valerio Scarani, Helle Bechmann-Pasquinucci, Nicolas J. Cerf, Miloslav Dušek, Norbert Lütkenhaus, and Momtchil Peev. The security of practical quantum key distribution. *Rev. Mod. Phys.*, 81:1301–1350, Sep 2009.

[19] Toyohiro Tsurumaru and Kiyoshi Tamaki. Security proof for quantum-key-distribution systems with threshold detectors. *Phys. Rev. A*, 78:032302, Sep 2008.

[20] Normand J. Beaudry, Tobias Moroder, and Norbert Lütkenhaus. Squashing models for optical measurements in quantum communication. *Phys. Rev. Lett.*, 101:093601, Aug 2008.

[21] O. Gittsovich, N. J. Beaudry, V. Narasimhachar, R. Romero Alvarez, T. Moroder, and N. Lütkenhaus. Squashing model for detectors and applications to quantum-key-distribution protocols. *Phys. Rev. A*, 89:012325, 2014.

[22] L. Trevisan. Extractors and pseudorandom generators. *Journal of the ACM*, 48(4):860–79, 2001.

[23] W. Mauerer, C. Portmann, and V. B. Scholz. A modular framework for randomness extraction based on Trevisan's construction. arXiv:1212.0520, 2012.

[24] P. Bierhorst, E. Knill, S. Glancy, Y. Zhang, A. Mink, S. Jordan, A. Rommal, Y.-K. Liu, B. Christensen, S. W. Nam, M. J. Stevens, and L. K. Shalm. Experimentally generated random numbers certified by the impossibility of superluminal signaling. *Nature*, 556:223–226, 2018.

[25] W. Hoeffding. Probability inequalities for sums of bounded random variables. *Journal of the American Statistical Association*, 58:13, 1963.
